# Impact of Drying Processes on Phenolics and In Vitro Health-Related Activities of Indigenous Plants in Thailand

**DOI:** 10.3390/plants11030294

**Published:** 2022-01-22

**Authors:** Pandaree Sirichai, Suwapat Kittibunchakul, Sirinapa Thangsiri, Nattira On-Nom, Chaowanee Chupeerach, Piya Temviriyanukul, Woorawee Inthachat, Onanong Nuchuchua, Amornrat Aursalung, Yuraporn Sahasakul, Somsri Charoenkiatkul, Uthaiwan Suttisansanee

**Affiliations:** 1 Institute of Nutrition, Mahidol University, Salaya, Phuttamonthon, Nakhon Pathom 73170, Thailand; riceberrii3@gmail.com (P.S.); suwapat.kit@mahidol.ac.th (S.K.); sirinapa.thang@outlook.com (S.T.); nattira.onn@mahidol.ac.th (N.O.-N.); chaowanee.chu@mahidol.ac.th (C.C.); piya.tem@mahidol.ac.th (P.T.); woorawee.int@mahidol.ac.th (W.I.); amornrat.aur@mahidol.ac.th (A.A.); yuraporn.sah@mahidol.ac.th (Y.S.); somsri.chr@mahidol.ac.th (S.C.); 2 National Nanotechnology Center (NANOTEC), National Science and Technology Development Agency (NSTDA), Klong Luang, Pathum Thani 12120, Thailand; onanong@nanotec.or.th

**Keywords:** wild edible plants, phenolics, antioxidant capacity, noncommunicable diseases, in vitro health properties, enzyme inhibition

## Abstract

Thailand has vast areas of tropical forests with many indigenous plants, but limited information is available on their phytochemical profile and in vitro inhibitions of enzymatic and nonenzymatic reactions. This study investigated phenolic profiles using liquid chromatography–electrospray ionization tandem mass spectrometry (LC–ESI-MS/MS), antioxidant activities, and in vitro inhibitory activities of 10 indigenous plants on key enzymes related to obesity (lipase), diabetes (α-amylase and α-glucosidase), and Alzheimer’s disease (cholinesterases and β-secretase). The nonenzymatic anti-glycation reaction was also investigated. The 10 indigenous plants were *Albizia lebbeck* (L.) Benth, *Alpinia malaccensis* (Burm.) Roscoe, *Careya arborea* Roxb., *Diplazium esculentum* (Retz.) Swartz, *Kaempferia roscoeana* Wall., *Millettia brandisiana* Kurz., *Momordica charantia*, *Phyllanthus* *emblica* L., *Zingiber cassumunar* Roxb, and *Zingiber citriodorum* J. Mood & T. Theleide. Preparations were made by either freeze-drying or oven-drying processes. Results suggested that the drying processes had a minor impact on in vitro inhibitions of enzymatic and nonenzymatic reactions (<4-fold difference). *P. emblica* was the most potent antioxidant provider with high anti-glycation activity (>80% inhibition using the extract concentration of ≤6 mg/mL), while *D. esculentum* effectively inhibited β-secretase activity (>80% inhibition using the extract concentration of 10 mg/mL). *C. arborea* exhibited the highest inhibitory activities against lipase (47–51% inhibition using the extract concentration of 1 mg/mL) and cholinesterases (>60% inhibition using the extract concentration of 2 mg/mL), while *Mi. brandisiana* dominantly provided α-amylase and α-glucosidase inhibitors (>80% inhibition using the extract concentration of ≤2 mg/mL). Information obtained from this research may support usage of the oven-drying method due to its lower cost and easier preparation step for these studied plant species and plant parts. Furthermore, the information on in vitro inhibitions of enzymatic and nonenzymatic reactions could be used as fundamental knowledge for further investigations into other biological activities such as cell culture or in vivo experiments of these health-beneficial plants.

## 1. Introduction

Thailand has many different types of forests that contain a wide variety of medicinal plants. Some are used as Food and Drug Administration (FDA)-approved supplements and are even available in markets, while others are limited to local medicinal applications due to limited scientific evidence. Many plants with uncertain biological properties are endangered as a result of improper agricultural management, while some remain unexplored. Therefore, the Plant Genetic Conservation Project under the royal initiation of Her Royal Highness Princess Maha Chakri Sirindhorn (RSPG) was established. This project promotes the sustainable conservation and allocation of plant resources to achieve full beneficial utilization through collaboration between researchers and communities. Knowledge gained through laboratory research is then transferred back to the community to increase the utility and proper management of the plants, eventually leading to sustainable forest conservation by the local communities.

Ten indigenous plants, namely, *Albizia lebbeck* (L.) Benth (*Alb. lebbeck*), *Alpinia malaccensis* (Burm.) Roscoe (*Alp. malaccensis*), *Careya arborea* Roxb. (*C. arborea*), *Diplazium esculentum* (Retz.) Swartz (*D. esculentum*), *Kaempferia roscoeana* Wall. (*K. roscoeana*), *Millettia brandisiana* Kurz. (*Mi. brandisiana*), *Momordica charantia* (*Mo. charantia*), *Phyllanthus emblica* L. (*P. emblica*), *Zingiber cassumunar* Roxb. (*Z. cassumunar*), and *Zingiber citriodorum* J. Mood & T. Theleide (*Z. citriodorum*), with frequent consumption and traditional health benefits, were collected from the plant genetic conservation areas under the RSPG project. The edible parts of these plants were dried with and without thermal treatment (oven-drying and freeze-drying processes, respectively) to investigate the effect of drying processes, while extraction of these dry plants was performed as tea infusions as a model for future food development, aiming for easy and convenient consumption. The 10 plant extracts were then investigated to determine their phenolics, antioxidant activities, and in vitro inhibitory properties of key enzymes associated with medicinal abilities against some noncommunicable diseases (NCDs). These enzymes included the lipase inhibitor associated with a reduction in lipid absorption for the treatment of obesity, inhibition of the carbohydrate-digesting enzymes, α-glucosidase and α-amylase, as an approach to control diabetes, and inhibition of the neurotransmitter-degrading enzymes, acetylcholinesterase (AChE) and butyrylcholinesterase (BChE), as well as the amyloid precursor protein (APP)-degrading enzyme β-secretase (BACE-1), in Alzheimer’s disease (AD). The inhibition of nonenzymatic reactions in the body that lead to cell deterioration (glycation reaction) also contribute to slow down the deterioration of the body and prevent NCDs in the long term. Knowledge gained from this study will provide valuable information on how to prepare plant samples under the studied conditions and their in vitro inhibitions of enzymatic and nonenzymatic reactions. Furthermore, the knowledge can be transferred back to the community. We hope that if the locals know that these wild plants have potential health benefits, the plants and their growth location (the forest) would be taken care of, leading to sustainable conservation. This, of course, will have to go along with proper agricultural management. Results will also support establishing appropriate management and utilization to prevent plant extinction, in line with the RSPG objectives.

## 2. Results

### 2.1. Phenolic Profiles

Phenolic profiles of the 10 indigenous plants were determined using liquid chromatography–electrospray ionization tandem mass spectrometry (LC–ESI-MS/MS), for which the parent ions (*m*/*z*) of the standards, collision energies, and radio frequencies (RF-lens) are shown in Table S1. Specificity, linearity, limit of detection (LOD), limit of quantification (LOQ), precision, and accuracy parameters are shown in Table S2. Calibration plots of all standards presented a linear relationship, with correlation coefficients (*R*^2^) higher than 0.99. LOD and LOQ values indicated the analytical method detection limits to quantify phenolic components in the samples. The percentage of relative standard deviation (%RSD) was obtained from the repeatability of peak areas within the same day, while the recovery percentage was within acceptable limits (80–120%). Results indicated that LC–ESI-MS/MS with selection reaction monitoring (SRM) was a suitable and reliable method for quantifying phenolics in the plant extracts. The LC–ESI-MS/MS chromatograms of the standards and plant extracts are shown in Figures S1 and S2, respectively.

Results from LC–ESI-MS/MS suggested that the 10 indigenous plants, namely, *Alb. lebbeck*, *Alp. malaccensis*, *C. arborea*, *D. esculentum*, *K. roscoeana*, *Mi. brandisiana*, *Mo. charantia*, *P. emblica*, *Z. cassumunar*, and *Z. citriodorum*, exhibited various flavonoids in different amounts (Table 1). *Alb. lebbeck* extract contained the greatest variety of flavonoids including quercetin, kaempferol, luteolin, apigenin, and isorhamnetin, with luteolin being the highest. Luteolin in *Alb. lebbeck* was also the highest amount detected among all plant extracts, while the second most abundant flavonoid apigenin was only detected in this plant. The highest kaempferol content was detected in *Z. cassumunar* extract, which also contained naringenin and isorhamnetin. The highest content of quercetin was detected in *C. arborea* extract, which also contained high amounts of kaempferol. Isorhamnetin was the highest in *K. roscoeana* extract, while *P. emblica* extract contained moderate amounts of quercetin and naringenin. Among all plant extracts, only minute amounts of flavonoids were detected in *D. esculentum* (quercetin, kaempferol, and luteolin) and *Mi. brandisiana* (quercetin, naringenin, luteolin, and isorhamnetin) extracts at less than 8 mg/100 g dry weight (DW), while *Z. citriodorum* extract possessed minute amounts of quercetin and luteolin but a moderate content of isorhamnetin. *Alp. malaccensis* extract contained only one flavonoid as isorhamnetin, while no flavonoids were detected in *Mo. charantia* extract.

Gallic acid and rosmarinic acid were the only phenolic acids detected in some indigenous plants (Table 1). High gallic acid contents were detected in *C. arborea* and *P. emblica* (fourfold higher than *C. arborea*), while a minute amount of rosmarinic acid was detected in *D. esculentum*.

Total phenolic contents (TPCs) of freeze-dried plant extracts ranged from 5.37–193.51 mg gallic acid equivalent (GAE)/g DW, while oven-dried extracts exhibited TPCs ranging from 5.15–184.82 mg GAE/g DW (Table 2). Both freeze-dried and oven-dried *P. emblica* extracts provided the highest TPCs among all plant extracts dried under the same process. *Z. citriodorum*, *Mo. Charantia*, and *Alp. malaccensis* recorded low TPCs (29–36-fold lower than *P. emblica*). Interestingly, most freeze-dried plant extracts exhibited only 1.0–1.2 times higher TPCs than their corresponding oven-dried extracts, except for *D. esculentum* with the freeze-dried extract being 3.4-fold higher than its oven-dried counterpart. The TPC of oven-dried *Mo. charantia* could not be measured due to limited plant quantity.

### 2.2. Antioxidant Capacities

The in vitro abilities to resist oxidative stress of the aqueous extracts prepared by freeze-drying and oven-drying from 10 indigenous plants in Thailand were investigated by 2,2-diphenyl-1-picrylhydrazyl (DPPH) radical-scavenging, ferric reducing antioxidant power (FRAP), and oxygen radical antioxidant capacity (ORAC) assays. The antioxidant activity of oven-dried *Mo. charantia* could not be measured due to limited plant quantity.

Plant extracts prepared by freeze-drying exhibited DPPH radical-scavenging activities ranging from 0.018–0.064 µmol Trolox equivalent (TE)/g DW, with *P. emblica* exhibiting the highest radical-scavenging activity and *D. esculentum* and *Mo. charantia* exhibiting the lowest (Table 3). A similar trend was observed in the FRAP assay, with reducing abilities of the 10 plant extracts ranging from 17.39–2101.53 µmol TE/g DW. The highest activity was observed in *P. emblica* and the lowest was observed in *Mo. charantia*. Likewise, antioxidant activities determined by the ORAC assay ranged from 83.98–2737.43 µmol TE/g DW. Again, the highest activity was found in *P. emblica*, while the lowest was detected in *Mo. charantia* and *Z. citriodorum*.

Similar results were observed in the oven-dried plant extracts (Table 3). The DPPH radical-scavenging activities of oven-dried plant extracts ranged from 0.020–0.063 µmol TE/g DW, while their FRAP and ORAC activities ranged from 16.07–2054.77 and 79.36–2465.73 µmol TE/g DW, respectively. *P. emblica* exhibited the highest antioxidant activities in all three assays, with results similar to freeze-dried plant extracts.

The effect of the drying process was assessed on antioxidant activities. The DPPH radical-scavenging activities of freeze-dried plant extracts were 1.0–1.3 times higher than oven-dried plant extracts and 1.0–1.9 times higher for FRAP activities. Freeze-dried plant extracts also exhibited ORAC activities 1.0–2.6 times higher than their oven-dried counterparts.

### 2.3. In Vitro Inhibitory Activities

The in vitro enzyme inhibitions of lipase, α-amylase, α-glucosidase, AChE, BChE, and BACE-1 were performed. Aqueous extracts from the 10 indigenous plants were prepared as freeze- and oven-dried samples to investigate the effect of heat treatment in the drying processes on plant biological activities. The quantity of oven-dried *Mo. charantia* was limited, and its enzyme-inhibitory activity could not be measured.

The inhibition of lipase, the lipid-degrading enzyme, could lead to lower fat absorption, resulting in one approach to obesity control [1]. The results indicated that all plant extracts exhibited lipase-inhibitory activities, with the exception of *Alb. lebbeck* (Table 4). The highest lipase inhibition was found in *C. arborea* (46.89% inhibition using extract concentration of 1 mg/mL), while the lowest was observed in *Mi. brandisiana* and *P. emblica* (5.57–6.79% inhibition using extract concentration of 10 mg/mL). Among the oven-dried plant extracts, *C. arborea* provided the highest inhibitory activity (51.27% using extract concentration of 1 mg/mL), with no inhibition observed in *Alb. lebbeck*. Interestingly, the oven-dried plant extracts exhibited 1.0–1.3 times higher lipase-inhibitory activities than freeze-dried samples. 

To control diabetes, inhibition of the carbohydrate-degrading enzymes, α-amylase and α-glucosidase, could result in low sugar production and, hence, lead to low absorption of sugar into serum [2]. The inhibition of these two carbohydrate-degrading enzymes was shown by all plant extracts except for *C. arborea* (Table 4). Results suggested that freeze-dried plant extracts of *D. esculentum* and *Mi. brandisiana* effectively inhibited α-amylase by more than 85% using an extract concentration of 2 mg/mL, while the lowest inhibitory activities were observed in freeze-dried plant extracts of *Alb. lebbeck* and *Alp. malaccensis* (less than 7% using an extract concentration of 10 mg/mL). Similar results were observed in oven-dried plant extracts, in which the highest inhibitions were observed in *D. esculentum* and *Mi. brandisiana* with 75.63% and 82.57% inhibition, respectively, using an extract concentration of 2 mg/mL. Comparing drying processes, the α-amylase-inhibitory activities of freeze-dried plant extracts were 1.0–1.4 times higher than their oven-dried counterparts.

For α-glucosidase inhibition, freeze- and oven-dried plant extracts of *D. esculentum* and *Mi. brandisiana* strongly inhibited the enzyme, with more than 95% inhibition using an extract concentration of 0.5 mg/mL (Table 4). Interestingly, *P. emblica* gave the third-highest inhibitory activities against both α-amylase and α-glucosidase. Freeze-dried plant extracts inhibited α-glucosidase 1.0–1.4 times more than oven-dried samples.

Two cholinesterases (AChE and BChE) and BACE-1 are targeted to ameliorate AD [3]. Cholinesterases are the enzymes that degrade the neurotransmitters, acetylcholines, used by cholinergic neurons. Loss of these neurons could cause attention and memory deficits, leading to AD distribution. BACE-1 is the enzyme degrading amyloid precursor protein (APP), leading to accumulation of amyloid plaques, another significant hallmark of neuropathological lesions in AD brain.

Results indicated that all plant extracts inhibited AChE and BChE reactions (Table 5). Interestingly, despite possessing no inhibition against α-amylase and α-glucosidase, both freeze- and oven-dried plant extracts of *C. arborea* exhibited the highest AChE-inhibitory activities (75.07% and 69.44% using an extract concentration of 2 mg/mL). Among the other freeze-dried plant extracts, *Mo. charantia* exhibited the lowest inhibitory activity (20.76%) using an extract concentration of 10 mg/mL. Comparing the drying processes, freeze-dried plant samples exhibited 1.0–1.2 times higher AChE-inhibitory activities than oven-dried samples.

Both freeze- and oven-dried *C. arborea* extracts also exhibited the highest BChE-inhibitory activities (69.64% and 62.46% using an extract concentration of 2 mg/mL), with the lowest BChE-inhibitory activities observed in *K. roscoeana* extract (Table 5). Freeze-dried plant extracts exhibited only 1.0–1.1 times higher BChE-inhibitory activities than oven-dried samples.

By contrast, only three plant extracts, *C. arborea*, *D. esculentum*, and *K. roscoeana* (both freeze- and oven-dried samples), exhibited BACE-1-inhibitory activities using an extract concentration of 10 mg/mL, while no BACE-1-inhibitory activity was detected in the remaining samples using the same extract concentration (Table 5). Interestingly, *D. esculentum*, with the strongest α-amylase and α-glucosidase inhibition, also exhibited the highest BACE-1-inhibitory activity (90.82% in freeze-dried and 83.70% in oven-dried samples), followed by *K. roscoeana* and *C. arborea*. Freeze-dried plant extracts exhibited 1.1–1.4 times higher inhibitory activities than oven-dried samples.

For nonenzymatic reactions, anti-glycation activities were also investigated using bovine serum albumin (BSA) induced by either sugar (d-glucose) or methylglyoxal (MG) as a model study (Table 6). Results indicated that plant extract samples prepared by freeze-drying exhibited anti-glycation activities induced by d-glucose ranging from 33.49–88.93% using an extract concentration of 2 mg/mL. Similar results were observed for plant sample extracts prepared by oven-drying. Anti-glycation activities induced by d-glucose ranged from 54.80–85.84% using the same extract concentration. Both freeze- and oven-dried plant extracts of *P. emblica* exhibited the highest anti-glycation activities (88.93% and 85.84%, respectively), while *Mo. charantia* gave the lowest. When comparing drying methods, extracts prepared from plant samples that passed through the freeze-drying process exhibited 1.0–1.2 times higher anti-glycation activities than those prepared by oven-drying.

For the glycation reaction induced by MG, freeze-dried plant extracts exhibited anti-glycation activities ranging from 22.79–85.57% using an extract concentration of 6 mg/mL, while oven-dried plant extracts ranged from 30.07–82.77% using the same extract concentration. Among all plant extracts, freeze- and oven-dried plant extracts of *P. emblica* and *C. arborea* gave the highest anti-glycation activities. Freeze-dried plant extracts exhibited 1.0–1.2 times higher anti-glycation activities than oven-dried samples.

### 2.4. Correlation Analysis by Principal Component Analysis (PCA)

Principle component analysis (PCA) was employed to study the relationships between plant extracts and various potential health benefits measured in vitro in a simple and more representative way. PCA correlates data better than a comparison between groups using R-squared analysis. Relationships between observations (freeze-dried or oven-dried plant extracts) and variables (TPCs, antioxidant activities (DPPH radical-scavenging, FRAP, and ORAC assays), enzyme-inhibitory activities (against lipase, α-amylase, α-glucosidase, AChE, BChE, and BACE-1 reactions), and anti-glycation properties) were determined using PCA. However, *Mo. Charantia* and its variables were excluded from the analysis because of limited sample quantities.

**Figure 1:**
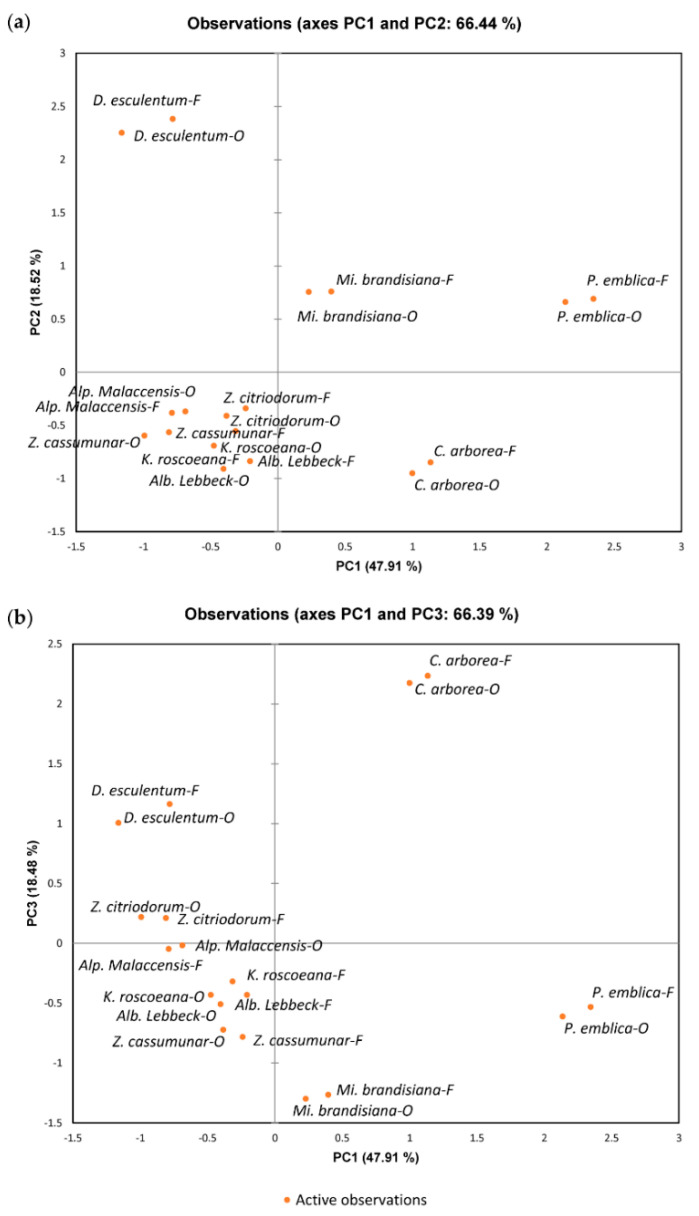
Principal component analysis (PCA) from mean values of tested variables including TPCs, antioxidant activities (determined by 2,2-diphenyl-1-picrylhydrazyl (DPPH) radical-scavenging, ferric reducing antioxidant power (FRAP), and oxygen radical antioxidant capacity (ORAC) assays), enzyme-inhibitory activities (against α-amylase, α-glucosidase, lipase, acetylcholinesterase (AChE), butyrylcholinesterase (BchE), and β-secretase (BACE-1)), and anti-glycation activities (methylglyoxal (MG) and sugar induction) (freeze-dried and oven-dried plant extracts): (**a**) observations between PC1 and PC2; (**b**) observations between PC1 and PC3.

Figure 1 shows the three axes (PC1, PC2, and PC3) that represented 84.92% of all variants, indicating a good portrayal of the total data. Remarkably, the same plant extract obtained by freeze-drying or oven-drying plotted close together, suggesting that the processing method played a minor role in the measured variables. 

**Figure 2:**
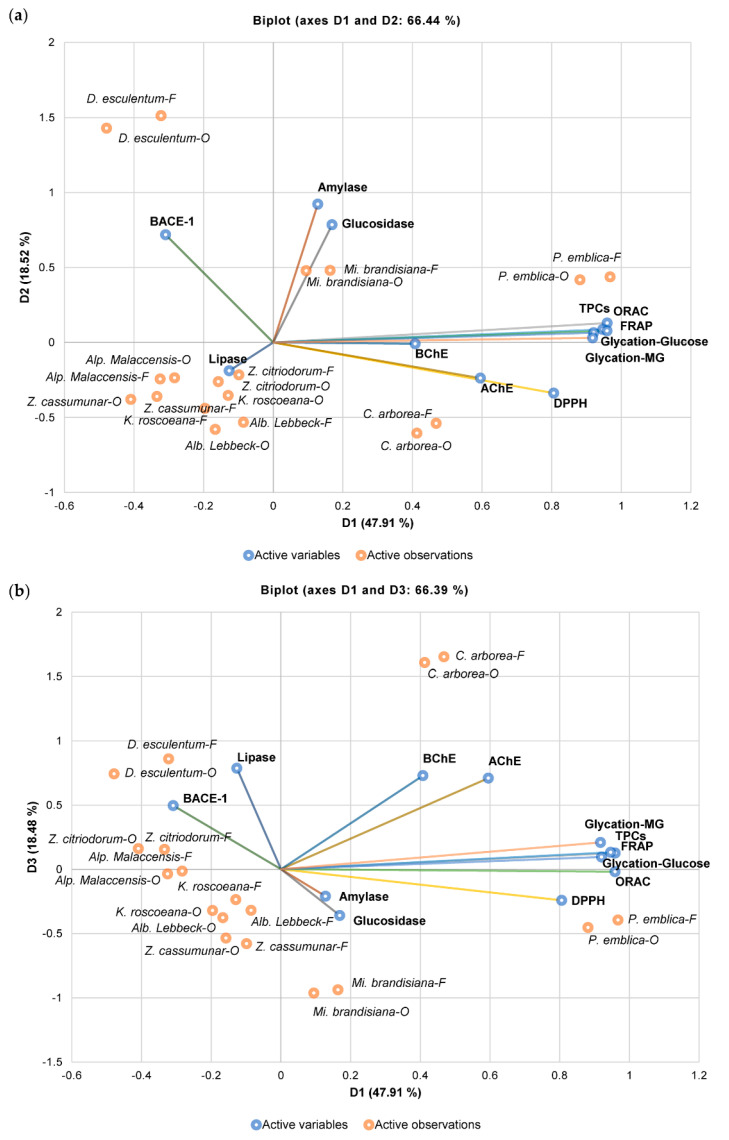
Principal component analysis (PCA) from mean values of tested variables including TPCs, antioxidant activities (determined by 2,2-diphenyl-1-picrylhydrazyl (DPPH) radical-scavenging, ferric reducing antioxidant power (FRAP), and oxygen radical antioxidant capacity (ORAC) assays), enzyme-inhibitory activities (against α-amylase, α-glucosidase, lipase, acetylcholinesterase (AChE), butyrylcholinesterase (BChE), and β-secretase (BACE-1)), and anti-glycation activities (methylglyoxal (MG) and sugar induction) of observations (freeze-dried and oven-dried plant extracts): (**a**) biplot between PC1 and PC2; (**b**) biplot between PC1 and PC3.

Figure 2 shows the biplot between observations and variables, consisting of three axes (PC1, PC2, and PC3). TPC, FRAP, and ORAC activities, as well as anti-glycation activities induced by sugar and MG, are presented on the PC1 axis, explaining 47.91% of the variance. The PC2 axis represents α-amylase-, α-glucosidase-, and BACE-1-inhibitory activities, explaining 18.52%, while the PC3 axis consists of DPPH radical-scavenging activities, as well as lipase-, AChE-, and BChE-inhibitory activities, covering 18.48% of the variance. Freeze-dried and oven-dried *P. emblica* exhibited the highest TPCs, antioxidant activities, and anti-glycation activities induced by both sugar and MG, and it formed a cluster close to these variables. The data also implied that TPCs contributed to antioxidant and anti-glycation activities observed in this sample. *D. esculentum* was projected close to BACE-1, while *Mi. brandisiana* was located close to α-amylase and α-glucosidase, suggesting their effective inhibitory functions against these enzymes. Moreover, *C. arborea* exhibited potent lipase-, AChE-, and BChE-inhibitory activities as it clustered with these variables.

## 3. Discussion

Thailand has a tropical climate, and many indigenous plants have potential health benefits, as suggested by a long history of traditional usage. However, the lack of scientific evidence concerning plant health properties has resulted in limited therapeutic applications and improper agricultural management. Some plants are at risk of extinction, and their potential health applications have not been recognized. To resolve this crisis, the Plant Genetic Conservation Project was established under the royal initiative of Her Royal Highness Princess Maha Chakri Sirindhorn (RSPG), with the ultimate goal of sustainable conservation and allocation of plant resources for optimized beneficial utilization. Ten indigenous plants, namely, *Alb. lebbeck*, *Alp. malaccensis*, *C. arborea*, *D. esculentum*, *K. roscoeana*, *Mi. brandisiana*, *Mo. charantia*, *P. emblica*, *Z. cassumunar*, and *Z. citriodorum*, were collected from the plant conservation area under the RSPG project to investigate their potential health benefits, which can lead to future food applications through proper plant handling. Commonly consumed parts of these plants, including shoots and young leaves (*Alb. Lebbeck*, *C. arborea*, *D. esculentum*, *K. roscoeana* and *Mi. brandisiana*), fruits (*Mo. Charantia* and *P. emblica*), and rhizomes (*Alp. malaccensis*, *Z. cassumunar* and *Z. citriodorum*), were prepared by freeze- and oven-drying and extracted as tea infusions before investigating antioxidant activities, which were determined by DPPH radical-scavenging, FRAP, and ORAC assays. Inhibitory activities against the key enzymes relevant to obesity (lipase), diabetes (α-glucosidase and α-amylase), and Alzheimer’s disease (AChE, BChE, and BACE-1) and the nonenzymatic glycation reaction were also assessed. These plant medicinal properties might result from the biological functions of their phenolics determined by LC–ESI-MS/MS. Findings suggested that the bioactivities of freeze- and oven-dried samples were similar, while slightly favoring the freeze-drying process. Among the 10 plant extracts, (i) *P. emblica* exhibited the highest antioxidant activities, TPCs, and anti-glycation activities, (ii) *D. esculentum* and *Mi. brandisiana* exhibited the highest inhibitory activities against carbohydrate-degrading enzymes, α-amylase and α-glucosidase, (iii) *D. esculentum* exhibited the strongest inhibitory activity against the key enzyme interfering with β-amyloid formation, BACE-1, and (iv) *C. arborea* exhibited the highest inhibitory activity against the fat-degrading enzyme, lipase, and the key enzymes controlling cholinergic neurotransmitters, AChE and BChE.

Interestingly, our results suggested that the drying processes (oven-drying at 60 °C for 5 h and freeze-drying at -50 °C and 0.086 mbar for 72 h) had minimal effects on plant biological properties. These results were valid only for our studied plant samples and their plant parts. Previous studies on thermal treatment of guava suggested that TPCs, total flavonoid contents (TFCs), and antioxidant activities determined by FRAP and ORAC assays of samples that underwent oven-drying (55 °C for 22 h) and freeze-drying (-50 °C and 0.025 mbar for 48 h) were insignificantly different [4]. An experiment using a tray dryer with suction flow on broccoli suggested that maximal antioxidant activities (through DPPH radical-scavenging assay) were achieved by high-temperature and short-time processes [5]. This conclusion was supported by a literature review on the effect of drying on antioxidant potential of fruits and vegetables [6]. However, this review also suggested that each fruit and vegetable under a particular thermal treatment possessed different results. For example, pear underwent thermal treatment of 54 °C with cold-air drying pretreatment, exhibiting reduced TPCs with increased antioxidant activities, while jujube underwent -50 °C for 48 h, exhibiting increased TPCs and reduced antioxidant activities [6]. Results from our experiments indicated little effect of drying methods on the biological activities of indigenous plants, supporting oven-drying processes with cheaper operational costs.

Among all plant extracts, our results indicated that a hot-water extract of *P. emblica* fruit exhibited high antioxidant activities, related to its high TPCs, especially gallic acid content. This experiment was aimed at investigating potential antioxidant activities using different mechanisms and the effect of drying processes on antioxidant activity. These antioxidant measurements underwent different mechanisms, in which the DPPH radical-scavenging and FRAP assays followed the single electron transfer (SET) mechanism, while the ORAC assay followed the hydrogen atom transfer (HAT) mechanism. Our results indicated that *P. emblica* extract exhibited higher ORAC values than DPPH radical-scavenging and FRAP activities, suggesting that this plant extract might contain antioxidants that follow the HAT rather than SET mechanism. Interestingly, previous studies suggested the effect of extraction solvent on antioxidant activities and TPCs of *P. emblica* fruit. The results on *P. emblica* fruit from China extracted with methanol, followed by partition with ethyl ether, ethyl acetate, butanol, and water, indicated that the highest TPCs and DPPH radical-scavenging activity were achieved in the ethyl acetate fraction, while those in the aqueous fraction were the lowest [7]. Extraction using hexane, ethyl acetate, and ethanol suggested that the ethanolic extract of *P. emblica* fruit from Indonesia provided the highest TPCs among all extracts [8]. However, comparing between ethanol and distilled water extraction, the aqueous extract of *P. emblica* fruit from India exhibited higher TPCs than its ethanolic extract [9], while high TPC and DPPH radical-scavenging activities were detected in methanolic extracts of *P. emblica* fruit from six regions of China [10]. A strong correlation between antioxidant activities and TPCs was also observed [9,10]. High antioxidant activity in *P. emblica* fruit resulted from the biological functions of its phenolics [11]. Phenolics in *P. emblica* fruit from China were geraniin, quercetin 3-β-d-glucopyranoside, isocorilagin, and kaempferol [7], while another group reported cinnamic acid, quercetin, 5-hydroxymethylfurfural, gallic acid, β-daucosterol, and ellagic acid [12]. The former used methanol extraction, followed by partition with ethyl acetate [7], while the latter employed 90% (*v*/*v*) ethanol extraction, followed by ethyl acetate [12]. Gallic acid, corilagin, and ellagic acid were also detected in the aqueous ethanolic extract of *P. emblica* fruits collected from 10 different habitats in China [13]. While our experiments found gallic acid as a predominant phenolic in *P. emblica*, it could be concluded that different growing locations yielded different types and quantities of phenolics in the plant samples. Even though, in previous reports, the plant samples were extracted using different solvents, as opposed to the aqueous extraction in our study, these reports found that antioxidative agents contributed to anti-glycation activities, while the ethanolic extracts of *P. emblica* fruit collected from Thailand and Saudi Arabia exhibited high antioxidant and anti-glycation activities [14,15]. Similar results were observed in the methanolic extract of *P. emblica* fruit from Sri Lanka, with the highest anti-glycation activity among nine antidiabetic plant extracts [16]. The most abundant phenolic detected in our *P. emblica*, gallic acid, was also able to inhibit advanced glycation end-products in both cell cultures and animal models [17,18,19].

Advanced glycation end-product formation is highly related to diabetic complications. Unlike *P. emblica* with high anti-glycation activities, *D. esculentum* and *Mi. brandisiana* were found to be the potent plants with high inhibitory activities against the key enzymes relevant to diabetic control (*P. emblica* was in third place). Prepared as tea infusions, the results of our in vitro experiments suggest potential antidiabetic activity through key enzyme inhibitions of *D. esculentum* and *Mi. brandisiana*. These results were supported by a recent review on *D. esculentum* [20], suggesting that the plant extract exhibited both α-amylase- and α-glucosidase-inhibitory activities at more than 50%, even though extraction condition was not reported. Furthermore, its hydroalcoholic extract (500 mg/kg) effectively halved serum glucose in streptozocin (STZ)-induced diabetic rats [21]. Upon being extracted by hot water (90 °C, 1 h), its α-glucosidase-inhibitory activity was eight times more effective than myricetin, suggesting that this plant extract was a potentially effective α-amylase and α-glucosidase inhibitor [22]. Several phenolics were isolated from *D. esculentum* including ascorbic acid, quercetin, cyanidins, *trans*-cinnamic acid, protocatechuic acid, and rutin [20,23], while we found quercetin, luteolin, and rosmarinic acid, which might be responsible for α-amylase- and α-glucosidase-inhibitory activities. It was previously suggested that quercetin exhibited the half-maximal inhibitory concentrations (IC_50_) of 500 and 7 µM against α-amylase and α-glucosidase, respectively, while those of luteolin were 360 and 21 µM against α-amylase and α-glucosidase, respectively [24]. Rosmarinic acid, on the other hand, was a less effective inhibitor against both carbohydrate-degrading enzymes [25]. Unfortunately, no information on the α-amylase- and α-glucosidase-inhibitory activities of *Mi. brandisiana* was previously reported, with the focus on bioactive compounds contained in its roots [26]. Only isoflavones and brandisianins were identified in the leaves of *Mi. brandisiana* [27,28]. However, we detected trace amounts of luteolin and isorhamnetin; thus, the anti-α-amylase and anti-α-glucosidase agents might be other phenolics.

*D. esculentum* strongly inhibited BACE-1, the key enzyme in β-amyloid formation, and one hypothesis underlying AD occurrence. Various pharmacological properties of *D. esculentum* were previously reported [29]; however, none concentrated on BACE-1 inhibition. Nevertheless, its aqueous leaf extract significantly elevated locomotor activity and stimulated the central nervous system (CNS) in mice [30], while a 70% (*v*/*v*) methanolic extract inhibited AChE with an IC_50_ value of 0.27 mg/mL [31], suggesting its potential as a source of anti-AD agents.

Interestingly, our results indicated that *C. arborea* effectively inhibited the fat-degrading enzyme, lipase. Only one previous report on lipase-inhibitory activity of *C. arborea* was available, albeit in the Korean language [32]. High lipase inhibition in *C. arborea* might be due to the biological function of kaempferol and quercetin since both were previously reported to be effective lipase inhibitors with IC_50_ values of approximately 0.23 mM [13,33]. The former acted as a competitive inhibitor, while the latter was a mixed type, close to a noncompetitive inhibitor [13,33]. In addition to its potential to reduce fat absorption, our results indicated that *C. arborea* is involved in controlling the degradation of cholinergic neurotransmitters through inhibitions of AChE and BChE. However, there are no previous reports on its AChE- and BChE-inhibitory properties. Nevertheless, the methanolic extract of its stem bark caused CNS depressant activity in Swiss albino mice and Wistar albino rats [34], while its abundant phenolics, kaempferol and quercetin, inhibited AChE with IC_50_ values of 3.05 and 3.60 µM, respectively [35]. The enzyme flavonoid inhibition constants (Ki) of kaempferol and quercetin against BChE were 92.8 and 38.3 µM, respectively [36], suggesting that kaempferol and quercetin are effective AChE and BChE inhibitors.

To summarize, our results revealed that drying processes, i.e., freeze-drying and oven-drying, of plants in this study played a minor role in the results of this experiment. Therefore, oven-drying would be a more appropriate method for locals due to its lower cost, instrumental availability, and easier and more convenient preparation step. We also found that *P. emblica* extract was the most potent antioxidant and anti-glycation provider, while *D. esculentum* extract was the most potent inhibitor against enzymes relevant to AD through β-amyloid formation. Furthermore, *D. esculentum* and *Mi. brandisiana* extracts were effective against enzymes relevant to diabetes, while *C. arborea* extract strongly inhibited enzymes relevant to obesity and AD through cholinergic degradation. Since hot-water extraction as a tea infusion was chosen as a model for easy and convenient preparation in our study, we hope that the knowledge gained from this research will be useful for future food development from these indigenous plants with high phenolics. Nevertheless, these in vitro findings need further testing in vivo and under clinical conditions, as in vitro studies do not cover the aspects of dose and the effect of bioavailability and pharmacokinetics. These plants, however, show potential for future pharmaceutical applications that will greatly benefit their sustainably, maintenance, advantageous utilization, and agricultural management in line with the RSPG objectives.

## 4. Materials and Methods

### 4.1. Sample Collection, Preparation, and Extraction

Ten indigenous plants, namely, *Alb. lebbeck*, *Alp. malaccensis*, *C. arborea*, *D. esculentum*, *K. roscoeana*, *Mi. brandisiana*, *Mo. charantia*, *P. emblica*, *Z. cassumunar*, and *Z. citriodorum*, were collected from the forest near Khuean Srinagarindra National Park, Tha Kra Dan sub-district, Si Sawat district, Kanchanaburi province, Thailand (14°38′22.5” N and 98°59′08.2” E), which is the conservative plant area under the Plant Genetic Conservation Project under the royal initiative of Her Royal Highness Princess Maha Chakri Sirindhorn (RSPG). All plant samples were deposited at Sireeruckhachati Nature Learning Park, Mahidol University, Nakhon Pathom, Thailand, and the voucher specimens were assigned as PBM-005664 (*Alb. lebbeck*), PBM-005663 (*Alp. malaccensis*), PBM-005656 (*C. arborea*), PBM-005654 (*D. esculentum*), PBM-005658 (*K. roscoeana*), PBM-005652 (*Mi. brandisiana*), PBM-005659 (*Mo. charantia*), PBM-005661 (*P. emblica*), PBM-005665 (*Z. cassumunar*), and PBM-005674 (*Z. citriodorum*). The edible parts, size, and physical appearances of all plants are shown in Table S3. 

The edible parts of the plant samples, including shoots and young leaves (*Alb. lebbeck*, *C. arborea*, *D. esculentum*, *K. roscoeana* and *Mi. brandisiana*), fruits (*Mo. charantia* and *P. emblica*), and rhizomes (*Alp. Malaccensis*, *Z. cassumunar* and *Z. citriodorum*), were cleaned and divided into two portions. The first was freeze-dried at -50 °C and 0.086 mbar for 72 h using a Heto powerdry PL9000 freeze-dryer (Heto Lab Equipment, Allerod, Denmark), while the other was dried at 60°C for 5 h using a hot air oven (electric convection dryer 12 kW/380 V, Kluay Num Thai, Bangkok, Thailand). Dry samples were ground using a Philips 600 W grinder (Philips Electronic Co., Ltd., Jakarta, Indonesia) into fine powder. The moisture contents were determined using a Halogen HE53 moisture analyzer (Mettler–Toledo AG, Greifensee, Switzerland), and the results were expressed as a percentage of moisture content, as shown in Table S4. The powdery samples were kept at -20 °C until analysis.

The extraction of both freeze-dried and oven-dried samples was performed as a tea infusion [37]. The powdery samples (1 g dry weight) were mixed with distilled water (100 mL) before incubating at 95 °C for 5 min using a temperature-controlled water bath shaker (WNE45 model from Memmert GmBh, Eagle, WI, USA). The supernatant was collected from centrifugation at 3800× *g* for 10 min using a refrigerated Hettich^®^ ROTINA 38R centrifuge (Andreas Hettich GmbH, Tuttlingen, Germany), and then filtered through a 0.45 µM polyethersulfone (PES) membrane syringe filter. The extracts were stored at -20 °C until analysis.

### 4.2. Determination of Phenolic Profiles Using Liquid Chromatography-Electrospray Ionization Tandem Mass Spectrometry

#### 4.2.1. Optimizing Reaction Monitoring Transitions of Authentic Standards

The molecular mass analysis of authentic standards of phenolics was performed using 12 phenolic acids and 12 flavonoids. Phenolic acid standards 3,4-dihydroxybenzoic acid (≥97% T), chlorogenic acid (>98.0% HPLC, T), 4–hydroxybenzoic acid (>99.0% GC, T), caffeic acid (>98.0% HPLC, T), syringic acid (>97.0% T), *p*-coumaric acid (>98.0% GC, T), ferulic acid (>98.0% GC, T), sinapic acid (>99.0% GC, T), and cinnamic acid (>98.0% HPLC) were from Tokyo Chemical Industry (Tokyo, Japan), while gallic acid (97.5–102.5% T), vanillic acid (≥97% HPLC), and rosmarinic acid (≥98% HPLC) were from Sigma–Aldrich (St. Louis, MO, USA). Flavonoid standards (-)-epigallocatechin gallate (>98.0% HPLC), hesperidin (>90.0% HPLC, T), myricetin (>97.0% HPLC), luteolin (>98.0% HPLC), quercetin (>98.0% HPLC, E), naringenin (>93.0% HPLC, T), kaempferol (>97.0% HPLC), apigenin (>98.0% HPLC), and genistein (>98.0% HPLC) were from Tokyo Chemical Industry (Tokyo, Japan), while isorhamnetin (≥99.0% HPLC) was from Extrasynthese (Genay, France), galangin (≥98.0% HPLC) was from Wuhan ChemFaces Biochemical Co., Ltd. (Hubei, China), and rutin (≥94% HPLC) was from Sigma–Aldrich (St. Louis, MO, USA). 

Each standard solution (10 µL of 20 µg/mL) was injected at a flow rate of 10 µL/min into a liquid chromatography-electrospray ionization tandem mass spectrometry (LC–ESI-MS/MS) system (a Dionex Ultimate 3000 series ultrahigh-performance liquid chromatography (UHPLC) system equipped with a diode array detector (DAD) from Thermo Fisher Scientific, Bremen, Germany). The experiments were operated by a TSQ Quantis Triple Quadrupole mass spectrometer (Thermo Fisher Scientific, Bremen, Germany) to obtain MS/MS ion fragments of authentic standards at specific collision energies and radio frequencies (RF-lens).

Negative and positive fragment ions were generated by a heated electrospray ion source (HESI) and measured by a TSQ Quantis Triple Quadrupole mass spectrometer. The MS parameters were as follows: mass range, 50–1000 *m*/*z*; positive ion, 3500 V, negative ion, 3500 V; sheath gas (N_2_), 30 Arb; auxiliary gas (N_2_), 15 Arb; ion transfer tube temperature, 325 °C; vaporizer temperature, 350 °C. Multiple mass spectrometric scanning modes, including full scanning (FS) and selective reaction monitoring (SRM), were selected for phenolic qualitative analysis. The data were further used in method validation and characterization of phenolics in the sample solutions by LC-ESI-MS/MS with *SRM mode*.

#### 4.2.2. Quantification of Phenolics

The LC–ESI-MS/MS with a Chromeleon 7 chromatography data system (version 7.2.9.11323 from Thermo Fisher Scientific, Bremen, Germany) was used for molecular mass analysis of 24 authentic standards of phenolics. A standard mixed solution containing all authentic phenolics was prepared and diluted in methanol at different levels for calibration curves and analytical method validations, as shown in Table S1. The chromatographic separation of the standards was carried out in an Accucore RP-MS column (2.1 mm × 100 mm, 2.6 μm, Thermo Fisher Scientific, Bremen, Germany) by eluting with a mobile phase composed of acetonitrile (elutent A) and 0.1% (*v/v*) formic acid in Milli-Q water (18.2 MΩ·cm resistivity at 25 °C) (eluent B). The gradient elution at 10% A and 90% B was performed at a flow rate of 0.5 mL/min for 10 min. The injection volume of the standard and sample solutions was 10 µL. The column temperature was kept at 35 °C. The collision energies of the authentic standards are shown in Table S1, and the chromatograms are shown in Figure S1.

#### 4.2.3. Method Validation

The validation of LC-ESI-MS/MS with SRM mode for quantifying phenolics in the samples followed the ICH Q2B guidelines (1997) [38] and works of Srinuanchai et al. (2019, 2021) [39,40], to observe parameters such as specificity, linear regression equation, correlation coefficient, limit of detection (LOD), limit of quantification (LOQ), precision, and accuracy (Table S2).

The selectivity and specificity analyses of phenolic detection in the samples were carried out using three SRM transitions of each phenolic at specific collision energies (V) and radiofrequencies (RF-lens; V).

Calibration curves of 24 authentic standards were prepared using the different concentrations of the stock standard solutions in methanol. The ranges of the standard concentrations were chosen depending on the range observed in the sample solutions. The calibration curves of each standard range were carried out in triplication on the same day. Linear plots (*y* = *ax* ± *c*) were presented between concentration ranges (*x*) and their ion peak areas (*y*). The correlation coefficients of the linear regressions were expressed as values of *R*^2^.

The determination of LOD and LOQ was calculated from the standard curve, which was analyzed in triplicate. LOD and LOQ were determined according to Equations (1) and (2).

LOD = (3.3σ)/S,(1)

LOQ = (10σ)/S,(2)

where σ is the standard deviation of the *Y*-intercept, and S is the average slope of the linear calibration curve.

Precision was determined using intra-day precision values of each standard, which were determined by seven replicate injections of a concentration level within the same day. The variations of retention time were presented as the percentage of relative standard deviation (%RSD).

Accuracy was determined using a known amount of the chemical standards, which was added to the sample solutions at three concentrations at low, medium, and high levels of linear calibration curves. The solutions of the spiked samples were injected into the LC–ESI-MS/MS with SRM system in triplicate. After that, the phenolic compounds in the spiked samples were quantified and calculated using Equation (3). The accuracy of this method was expressed as percentage recovery (%recovery) using the following equation:

%recovery = [(observed amount - initial amount) × 100]/spiked amount.(3)

#### 4.2.4. Sample Preparations

The powdery sample (0.5 g) was dissolved in a mixture of formic acid (40 mL) and 62.5% (*v/v*) methanol containing 0.5 g *tert*-butylhydroquinone (TBHQ) (10 mL) before shaking in a temperature-controlled water bath shaker (TW20 series from Julabo GmbH, Seelbach, Germany) at 80 °C for 2 h. The mixture was then put on ice for 5 min to stop the reaction. To the mixture, 1% (*v/v*) ascorbic acid (100 µL) was added before sonicating in an ultrasonic bath (Branson Ultrasonics™ M series, Branson Ultrasonics Corp., Danbury, CT, USA) for 5 min. The volume of the mixture was adjusted to 50 mL with 62.5% (*v/v*) methanol containing 0.5 g TBHQ before filtering through a 0.22 µM polytetrafluoroethylene (PTFE) membrane syringe filter and injecting into the LC–ESI-MS/MS system as described above.

### 4.3. Determination of Total Phenolic Contents

Total phenolic contents (TPCs) were evaluated as previously described [41]. The presence of phenolics was detected using Folin–Ciocâlteu’s phenol reagent on a Synergy^TM^ HT 96-well UV–visible microplate reader (BioTek Instruments, Inc., Winooski, VT, USA) with Gen 5 data analysis software at 765 nm. The TPCs were calculated using a standard curve of gallic acid (0–200 µg/mL) and expressed as mg gallic acid equivalent (GAE)/g dry weight (DW).

### 4.4. Determination of Antioxidant Activities

The determination of antioxidant activities was performed as previously described [42] using 2,2-diphenyl-1-picrylhydrazyl (DPPH) radical-scavenging, ferric ion reducing antioxidant power (FRAP), and oxygen radical absorbance capacity (ORAC) assays to cover both antioxidant mechanisms (single electron transfer and hydrogen atom transfer mechanisms). The DPPH radical-scavenging assay was performed using the DPPH reagent on a 96-well UV–visible microplate reader at 520 nm. The DPPH radical-scavenging activity was evaluated using a standard curve of Trolox, a water-soluble vitamin E analogue (0.01–0.64 mM). The FRAP assay was detected using FRAP reagent at 595 nm, while the ORAC assay was monitored using fluorescein reagent at an excitation wavelength of 485 nm and emission wavelength of 528 nm. The FRAP activity was evaluated using the Trolox standard of 7.81–250.00 µM, while ORAC activity used the Trolox standard of 3.12–100.00 µM. The antioxidant activities of all three assays were expressed as µmol Trolox equivalent (TE)/g dry weight (DW).

### 4.5. Enzyme-Inhibitory Activities

In vitro biological activities of the plant extracts against some noncommunicable diseases through inhibition of some key enzymes were determined using lipase (obesity), α-amylase and α-glucosidase (diabetes), and acetylcholinesterase, butyrylcholinesterase, and β-secretase (Alzheimer’s disease). Furthermore, nonenzymatic reaction of anti-glycation induced by both d-glucose and methylglyoxal (MG) was also investigated. According previous well-established protocols [43,44,45,46], the enzymatic and nonenzymatic assays were modified as briefly described below.

The lipase-inhibitory assay was composed of 100 µL of 5 µg/mL *Candida rugosa* lipase (type VII, ≥700 unit/mg), 10 µL of 16 mM 5,5′-dithiobis(2-nitrobenzoic acid) (DTNB), 50 µL of 0.2 mM 5,5′-dithiobis(2-nitrobenzoic-*N*-phenacyl-4,5-dimethyyhiazolium bromide), and 40 µL of the extract. The inhibitory activity was calculated as a decline in enzyme kinetics at 412 nm.

The α-amylase-inhibitory assay was composed of 100 µL of 20 mg/mL porcine pancreatic α-amylase (type VII, ≥10 unit/mg), 50 µL of 30 mM *p*-nitrophenyl-α-d-maltopentaoside, and 50 µL of the extract. Similarly, the α-glucosidase-inhibitory assay was composed of 100 µL of 1 U/mL *Saccharomyces cerevisiae* α-glucosidase (type I, ≥10 U/mg protein), 50 µL of 2 mM *p*-nitrophenyl-α-d-glucopyranoside, and 50 µL of the extract. The inhibitory activities of both enzyme assays were calculated as a decline in enzyme kinetics at 405 nm.

The acetylcholinesterase (AChE)-inhibitory assay was composed of 100 μL of 20 ng *Electrophorus electricus* AChE (1000 units/mg), 10 µL of 16 mM DTNB, 40 μL of 0.8 mM acetylthiocholine (Ach), and 40 µL of the extract. Likewise, the butyrylcholinesterase (BChE)-inhibitory assay was composed of 100 µL of 0.5 µg/mL equine serum BChE (≥10 units/mg), 10 µL of 16 mM DTNB, 40 µL of 0.4 mM butyrylthiocholine (BCh), and 40 µL of the extract. The inhibitory activities of both enzyme assays were calculated as a decline in enzyme kinetics at 412 nm.

The β-secretase (BACE-1)-inhibitory activity was investigated using a BACE-1 fluorescence resonance energy transfer (FRET) assay kit (Sigma-Aldrich, St. Louis, MO, USA) according to the manufacturer’s recommendations. The inhibitory activity was determined using an excitation wavelength (λ_ex_) at 320 nm and an emission wavelength (λ_em_) at 405 nm as an end-point assay. 

The anti-glycation reaction induced by d-glucose was composed of 50 µL of 20 mg/mL bovine serum albumin (BSA, ≥98.0% agarose gel electrophoresis) in 100 mM potassium phosphate buffer (pH 7.4) containing 0.02% (*w/v*) sodium azide, 25 µL of 1 M d-glucose, and 25 µL of extract. For the anti-glycation reaction induced by methylglyoxal (MG), 25 µL of 4 mM MG was used instead of d-glucose. The reaction mixture was incubated at 37 °C for 3 weeks in the dark. Inhibitory activity was evaluated using λ_ex_ 330 nm and λ_em_ 410 nm as an end-point assay.

All chemicals and reagents were received from Sigma-Aldrich (St. Louis, MO, USA). The inhibitory percentage was calculated using the following equation.

(4) $$\%\;\mathrm{inhibition}=\left(1-\frac{B-b}{A-a}\right)\;\times \;100,$$

where *A* is the initial velocity of the control reaction with enzyme (control), *a* is the initial velocity of the control reaction without enzyme (control blank), *B* is the initial velocity of the enzyme reaction with extract (sample), and *b* is the initial velocity of the reaction with extract but without enzyme (sample blank). The inhibitory percentage of BACE-1 and anti-glycation reactions was calculated using the same equation but changing from initial velocity to absorbance at a particular wavelength.

### 4.6. Statistical Analysis

All experiments were performed in triplicate (*n* = 3), and the results were expressed as the mean ± standard deviation (SD). The significant differences between values at *p* < 0.05 were determined according to one-way analysis of variance (ANOVA) followed by Duncan’s multiple comparison test (more than two data) and unpaired *t*-test (two data) using the statistical package for the social sciences (version 18 for Windows, SPSS Inc., Chicago, IL, USA).

Principal component analyses (PCAs) of TPCs, antioxidant activities, and enzymatic and nonenzymatic inhibitory activities of freeze-dried and oven-dried plant extracts were evaluated using XLSTAT^®^ Trial (Addinsoft Inc., New York, NY, USA). 

## Figures and Tables

**Table 1:** Phenolic profiles of 10 indigenous plant extracts determined using liquid chromatography-electrospray ionization tandem mass spectrometry (LC–ESI-MS/MS).

Samples	Phenolic Profile (mg/100 g Dry Weight)							
	Flavonoids						Phenolic Acids	
	Quercetin	Kaempferol	Naringenin	Luteolin	Apigenin	Isorhamnetin	Gallic Acid	Rosmarinic Acid
*Alb. lebbeck*	61.87 ± 1.83 ^c, C^	36.39 ± 3.30 ^c,D^	ND	134.20 ± 8.00 ^a,A^	105.11 ± 7.63 ^a,B^	8.05 ± 0.27 ^d,E^	ND	ND
*Alp. malaccensis*	ND	ND	ND	ND	ND	0.58 ± 0.03 ^e,A^	ND	ND
*C. arborea*	151.35 ± 8.29 ^a,B^	212.73 ± 10.22 ^b,A^	ND	ND	ND	ND	63.65 ± 1.63 ^b,C^	ND
*D. esculentum*	1.81 ± 0.10 ^e,C^	2.21 ± 0.16 ^e,C^	ND	7.36 ± 0.42 ^b,B^	ND	ND	ND	13.27 ± 0.49 ^A^
*K. roscoeana*	135.45 ± 2.57 ^b,B^	15.46 ± 0.73 ^d,C^	ND	ND	ND	229.64 ± 1.83 ^a,A^	ND	ND
*Mi. brandisiana*	<LOD	ND	<LOD	6.40 ± 0.08 ^b,A^	ND	0.37 ± 0.02 ^e,B^	ND	ND
*Mo. charantia*	ND	ND	ND	ND	ND	ND	ND	ND
*P. emblica*	18.97 ± 0.07 ^d,B^	4.51 ± 0.45 ^e,C^	9.21 ± 0.84 ^b,C^	ND	ND	ND	252.55 ± 13.65 ^a,A^	ND
*Z. cassumunar*	ND	237.09 ± 8.15 ^a,A^	15.73 ± 1.10 ^a,B^	ND	ND	15.16 ± 0.19 ^c,B^	ND	ND
*Z. citriodorum*	0.98 ± 0.06 ^e,C^	ND	ND	6.41 ± 0.08 ^b,B^	ND	25.00 ± 1.19 ^b,A^	ND	ND

All data are expressed as the mean ± standard deviation (SD) of triplicate experiments (*n* = 3). Lowercase letters indicate significantly different contents of the same phenolic in different plant extracts, while capital letters indicate significantly different contents of different phenolics in the same plant extracts at *p* < 0.05 using one-way analysis of variance (ANOVA) and Duncan’s multiple comparison test. ND: not detected; LOD: limit of detection (as shown in Table S2).

**Table 2:** Total phenolic contents (TPCs) of 10 indigenous plant extracts prepared from freeze-drying and oven-drying processes.

Samples	Total Phenolic Contents (mg GAE/g Dry Weight)	
	Freeze-Dried	Oven-Dried
*Alb. lebbeck*	28.89 ± 0.61 ^d,^*	24.18 ± 0.64 ^D^
*Alp. malaccensis*	6.68 ± 0.38 ^g,^*	6.12 ± 0.26 ^H^
*C. arborea*	107.00 ± 3.73 ^b,^*	96.69 ± 1.31 ^B^
*D. esculentum*	30.08 ± 0.56 ^d,^*	8.91 ± 0.13 ^F,G^
*K. roscoeana*	15.36 ± 0.24 ^e,^*	14.86 ± 0.34 ^E^
*Mi. brandisiana*	39.10 ± 0.71 ^c,^*	34.69 ± 0.90 ^C^
*Mo. charantia*	6.11 ± 0.11 ^g^	NA
*P. emblica*	193.51 ± 6.59 ^a,^*	184.82 ± 5.91 ^A^
*Z. cassumunar*	10.11 ± 0.45 ^f,^*	9.55 ± 0.21 ^F^
*Z. citriodorum*	5.37 ± 0.10 ^g,^*	5.15 ± 0.06 ^I^

All data are expressed as the mean ± standard deviation (SD) of triplicate experiments (*n* = 3). Lowercase and capital letters indicate significantly different total phenolic contents of freeze-dried and oven-dried plant extracts, respectively, at *p* < 0.05 using one-way analysis of variance (ANOVA) and Duncan’s multiple comparison test; * significantly different total phenolic contents at *p* < 0.05 between freeze-dried and oven-dried plant extracts in the same measurements according to an unpaired *t*-test. GAE: gallic acid equivalent; NA: not available.

**Table 3:** Antioxidant activities of 10 indigenous plant extracts that prepared from freeze-drying and oven-drying processes.

Samples	Antioxidant Activities (µmol TE/g Dry Weight)					
	DPPH Radical-Scavenging Assay		FRAP Assay		ORAC Assay	
	Freeze-Dried	Oven-Dried	Freeze-Dried	Oven-Dried	Freeze-Dried	Oven-Dried
*Alb. lebbeck*	0.055 ± 0.001 ^d,^*	0.052 ± 0.001 ^D^	135.32 ± 2.58 ^d,^*	102.53 ± 1.92 ^D^	516.84 ± 90.12 ^c,d^	473.85 ± 61.77 ^C^
*Alp. malaccensis*	0.039 ± 0.001 ^f,^*	0.038 ± 0.001 ^G^	43.05 ± 0.61 ^h,^*	41.82 ± 0.49 ^F^	196.78 ± 15.90 ^f,^*	166.01 ± 3.49 ^E^
*C. arborea*	0.059 ± 0.001 ^b,^*	0.059 ± 0.000 ^B^	1078.32 ± 66.18 ^b,^*	1005.69 ± 33.96 ^B^	1218.98 ± 151.56 ^b^	1077.60 ± 130.48 ^B^
*D. esculentum*	0.018 ± 0.001 ^h,^*	0.014 ± 0.001 ^I^	75.57 ± 1.98 ^g,^*	40.30 ± 1.22 ^F^	236.87 ± 30.76 ^f,^*	89.42 ± 5.52 ^F^
*K. roscoeana*	0.045 ± 0.001 ^e,^*	0.042 ± 0.001 ^F^	109.11 ± 2.77 ^e,^*	99.12 ± 2.28 ^D^	509.56 ± 8.02 ^c,d^	489.36 ± 39.46 ^C^
*Mi. brandisiana*	0.058 ± 0.001 ^c,^*	0.056 ± 0.001 ^C^	197.12 ± 3.14 ^c,^*	166.44 ± 2.21 ^C^	554.78 ± 69.21 ^c^	517.55 ± 54.95 ^C^
*Mo. charantia*	0.018 ± 0.001 ^h^	NA	30.93 ± 0.91 ^h,i^	NA	86.35 ± 6.23 ^g^	NA
*P. emblica*	0.064 ± 0.001 ^a,^*	0.063 ± 0.000 ^A^	2101.53 ± 37.10 ^a,^*	2054.77 ± 23.20 ^A^	2737.43 ± 191.95 ^a,^*	2465.73 ± 166.60 ^A^
*Z. cassumunar*	0.045 ± 0.001 ^e^	0.044 ± 0.000 ^E^	78.00 ± 1.82 ^f^	76.46 ± 1.82 ^E^	349.18 ± 35.02 ^e^	325.26 ± 18.50 ^D^
*Z. citriodorum*	0.022 ± 0.001 ^g,^*	0.020 ± 0.001 ^H^	17.39 ± 0.44 ^i,^*	16.07 ± 0.24 ^G^	83.98 ± 11.19 ^g^	79.36 ± 6.06 ^F^

All data are expressed as the mean ± standard deviation (SD) of triplicate experiments (*n* = 3). Lowercase and capital letters indicate significantly different antioxidant activities of freeze-dried and oven-dried plant extracts, respectively, at *p* < 0.05 using one-way analysis of variance (ANOVA) and Duncan’s multiple comparison test; * significantly different antioxidant activities at *p* < 0.05 between freeze-dried and oven-dried plant extracts in the same measurements according to an unpaired *t*-test. DPPH: 2,2-diphenyl-1-picrylhydrazyl; FRAP: ferric ion reducing antioxidant power; ORAC: oxygen radical absorbance capacity; TE: Trolox equivalent; NA: not available.

**Table 4:** In vitro inhibitory activities of 10 indigenous plant extracts prepared from freeze- and oven-drying processes against the key enzymes relevant to obesity (lipase) and diabetes (α-amylase and α-glucosidase).

Samples	Inhibitory Activity (%)					
	^1^ Lipase		^2^ α–Amylase		^3^ α–Glucosidase	
	Freeze-Dried	Oven-Dried	Freeze-Dried	Oven-Dried	Freeze-Dried	Oven-Dried
*Alb. lebbeck*	ND	ND	6.79 ± 0.70 ^e,^*	4.80 ± 0.47 ^E^	25.88 ± 2.04 ^c,^*	19.10 ± 1.38 ^C^
*Alp. malaccensis*	82.92 ± 2.70 ^a^	84.03 ± 0.15 ^A^	6.80 ± 0.07 ^e^	6.59 ± 0.90 ^D^	81.41 ± 0.55 ^a^	80.90 ± 0.78 ^A^
*C. arborea*	46.89 ± 4.45 ^#^	51.27 ± 4.16 ^#^	ND	ND	ND	ND
*D. esculentum*	45.75 ± 3.05 ^b^	47.49 ± 1.73 ^B^	85.35 ± 9.02 ^#^	75.63 ± 8.22 ^#^	97.63 ± 0.17 ^#,^*	95.03 ± 0.96 ^#^
*K. roscoeana*	34.73 ± 3.70 ^c^	36.20 ± 2.45 ^C^	9.88 ± 1.28 ^d^	8.90 ± 1.24 ^C^	5.48 ± 0.64 ^f^	ND
*Mi. brandisiana*	6.79 ± 0.74 ^e,^*	8.21 ± 0.91 ^E^	87.65 ± 7.30 ^#^	82.57 ± 5.10 ^#^	96.56 ± 0.28 ^#,^*	95.05 ± 1.24 ^#^
*Mo. charantia*	37.22 ± 2.46 ^c^	NA	15.87 ± 1.56 ^c^	NA	19.12 ± 1.22 ^d^	NA
*P. emblica*	5.57 ± 0.59 ^e,^*	7.39 ± 0.92 ^E^	61.30 ± 2.68 ^a^	59.16 ± 1.67 ^A^	81.86 ± 3.23 ^##,^*	77.12 ± 3.16 ^##^
*Z. cassumunar*	20.60 ± 2.30 ^d,^*	25.69 ± 3.03 ^D^	19.26 ± 1.63 ^b^	18.11 ± 1.50 ^B^	63.05 ± 0.93 ^b,^*	57.25 ± 0.56 ^B^
*Z. citriodorum*	34.82 ± 2.28 ^c^	36.35 ± 0.78 ^C^	10.07 ± 0.95 ^d^	9.15 ± 0.91 ^C^	11.61 ± 1.02 ^e,^*	9.29 ± 1.01 ^D^

All data are expressed as the mean ± standard deviation (SD) of triplicate experiments (*n* = 3). Lowercase and capital letters indicate significantly different inhibitory activities of freeze-dried and oven-dried plant extracts, respectively, at *p* < 0.05 using one-way analysis of variance (ANOVA) and Duncan’s multiple comparison test; * significantly different inhibitory activities at *p* < 0.05 between freeze- and oven-dried plant extracts in the same enzyme assay according to an unpaired *t*-test. ^1^ Final concentration of all extracts was 10 mg/mL except ^#^, which indicated a final concentration of 1 mg/mL; ^2^ final concentration of all extracts was 10 mg/mL except ^#^, which indicated a final concentration of 2 mg/mL; ^3^ final concentration of all extracts was 10 mg/mL except ^#^, which indicated a final concentration of 0.5 mg/mL, and ^##^, which indicated a final concentration of 1 mg/mL. ND: not detected; NA: not available.

**Table 5:** In vitro inhibitory activities of 10 indigenous plant extracts prepared from freeze- and oven-drying processes against the key enzymes relevant to Alzheimer’s disease: acetylcholinesterase (AChE), butyrylcholinesterase (BChE), and β–secretase (BACE–1).

Samples	Inhibitory Activity (%)					
	^1^ AChE		^1^ BChE		^1^ BACE-1	
	Freeze-Dried	Oven-Dried	Freeze-Dried	Oven-Dried	Freeze-Dried	Oven-Dried
*Alb. lebbeck*	40.72 ± 1.86 ^b,^*	38.10 ± 2.22 ^B^	74.67 ± 1.92 ^a,b,^*	68.77 ± 1.93 ^B^	ND	ND
*Alp. malaccensis*	34.43 ± 1.43 ^c^	33.64 ± 1.88 ^C^	45.12 ± 1.69 ^e,d^	42.46 ± 3.10 ^C^	ND	ND
*C. arborea*	75.07 ± 1.53 ^#,^*	69.44 ± 1.86 ^#^	69.64 ± 2.96 ^#,^*	62.46 ± 2.27 ^#^	18.11 ± 1.28 ^c,^*	12.81 ± 0.37 ^C^
*D. esculentum*	33.15 ± 2.18 ^c,^*	31.27 ± 2.44 ^D^	74.70 ± 1.91 ^a,b,^*	68.02 ± 2.25 ^B^	90.82 ± 0.18 ^a,^*	83.70 ± 1.10 ^A^
*K. roscoeana*	27.12 ± 1.37 ^e,^*	22.10 ± 1.17 ^G^	37.19 ± 2.37 ^f^	35.81 ± 2.17 ^D^	25.23 ± 1.46 ^b^	17.46 ± 3.29 ^B^
*Mi. brandisiana*	29.56 ± 1.22 ^d,^*	28.08 ± 1.57 ^E^	46.30 ± 2.49 ^d,^*	43.87 ± 2.09 ^C^	ND	ND
*Mo. charantia*	20.76 ± 1.39 ^g^	NA	55.10 ± 0.89 ^c^	NA	ND	NA
*P. emblica*	53.84 ± 3.45 ^a,^*	46.95 ± 2.45 ^A^	76.20 ± 2.32 ^a,^*	73.95 ± 1.37 ^A^	ND	ND
*Z. cassumunar*	30.51 ± 1.36 ^d^	29.57 ± 0.93 ^D,E^	44.44 ± 0.17 ^e,d,^*	43.49 ± 0.99 ^C^	ND	ND
*Z. citriodorum*	25.28 ± 0.77 ^f,^*	24.01 ± 1.23 ^F^	73.85 ± 1.20 ^b^	72.77 ± 2.82 ^A^	ND	ND

All data are expressed as the mean ± standard deviation (SD) of triplicate experiments (*n* = 3). Lowercase and capital letters indicate significantly different inhibitory activities of freeze- and oven-dried plant extracts, respectively, at *p* < 0.05 using one-way analysis of variance (ANOVA) and Duncan’s multiple comparison test; * significantly different inhibitory activities at *p* < 0.05 between freeze- and oven-dried plant extracts in the same enzyme assay according to an unpaired *t*-test. ^1^ Final concentration of all extracts was 10 mg/mL except ^#^, which indicated a final concentration of 2 mg/mL. ND: not detected; NA: not available.

**Table 6:** Anti-glycation activities of 10 indigenous plant extracts prepared from freeze-drying and oven-drying processes.

Samples	Anti-Glycation Reaction (%Inhibition)			
	^1^d-Glucose Induction		^2^ Methylglyoxal Induction	
	Freeze-Dried	Oven-Dried	Freeze-Dried	Oven-Dried
*Alb. lebbeck*	60.89 ± 1.46 ^g,h,^*	55.65 ± 1.25 ^G^	33.57 ± 0.59 ^g,f,^*	30.07 ± 0.85 ^E^
*Alp. malaccensis*	62.14 ± 0.62 ^g,^*	58.53 ± 0.55 ^F^	33.85 ± 1.11 ^g,f,^*	31.78 ± 0.36 ^E^
*C. arborea*	84.28 ± 1.36 ^b^	81.19 ± 1.68 ^B^	83.67 ± 0.18 ^a,^*	80.56 ± 0.61 ^A^
*D. esculentum*	66.41 ± 0.10 ^e,f,^*	54.80 ± 1.37 ^G^	42.79 ± 2.96 ^e,^*	35.04 ± 1.62 ^D^
*K. roscoeana*	71.13 ± 1.84 ^d,^*	65.63 ± 1.48 ^D^	54.10 ± 0.90 ^c,^*	49.40 ± 0.51 ^C^
*Mi. brandisiana*	77.82 ± 0.68 ^c,^*	74.39 ± 1.23 ^C^	59.88 ± 2.19 ^b,^*	53.09 ± 3.14 ^B^
*Mo. charantia*	33.49 ± 1.10 ^i^	NA	22.79 ± 0.80 ^h^	NA
*P. emblica*	88.93 ± 0.49 ^a,^*	85.84 ± 0.18 ^A^	85.57 ± 1.17 ^a,^*	82.77 ± 0.36 ^A^
*Z. cassumunar*	68.05 ± 2.53 ^e,^*	62.35 ± 1.70 ^E^	51.39 ± 2.97 ^c,d^	49.22 ± 2.78 ^C^
*Z. citriodorum*	64.67 ± 0.47 ^f,^*	57.01 ± 4.30 ^F,G^	34.86 ± 0.49 ^f,^*	30.96 ± 2.23 ^E^

All data are expressed as the mean ± standard deviation (SD) of triplicate experiments (*n* = 3). Lowercase and capital letters indicate significantly different inhibitory activities of freeze-dried and oven-dried plant extracts, respectively, at *p* < 0.05 using one-way analysis of variance (ANOVA) and Duncan’s multiple comparison test; * significantly different inhibitory activities at *p* < 0.05 between freeze-dried and oven-dried plant extracts in the same enzyme assay according to an unpaired *t*-test. ^1^ Final concentration of all extracts was 2 mg/mL; ^2^ final concentration of all extracts was 6 mg/mL; NA: not available.

## Data Availability

Data are contained within this article and the Supplementary Materials.

## Supplementary Materials

The following supporting information can be downloaded at www.mdpi.com/article/10.3390/plants11030294/s1: Table S1. Fragment ions of 24 authentic standards of phenolics using liquid chromatography-electrospray ionization tandem mass spectrometry (LC–ESI-MS/MS) in selective reaction monitoring (SRM) mode; Table S2. The validation parameters of 24 authentic standards of phenolics using liquid chromatography-electrospray ionization tandem mass spectrometry (LC–ESI-MS/MS) in selective reaction monitoring (SRM) mode; Table S3. The scientific name, voucher specimen, folk medicine, plant biology and characterization, and physical appearance of the edible part of the plant samples; Table S4. The percentage of moisture content in edible part of fresh, freeze-dried, and oven-dried plant samples; Figure S1. The liquid chromatography-electrospray ionization tandem mass spectrometry (LC–ESI-MS/MS) chromatograms of 24 authentic standards of phenolics; Figure S2. The liquid chromatography-electrospray ionization tandem mass spectrometry (LC–ESI-MS/MS) chromatograms of 10 plant samples: (a) *Albizia lebbeck* (L.) Benth, (b) *Alpinia malaccensis* (Burm.) Roscoe, (c) *Careya arborea* Roxb., (d) *Diplazium esculentum* (Retz.) Swartz, (e) *Kaempferia roscoeana* Wall., (f) *Millettia brandisiana* Kurz., (g) *Momordica charantia*, (h) *Phyllanthus emblica* L., (i) *Zingiber cassumunar* Roxb., and (j) *Zingiber citriodorum* J. Mood & T. Theleide.
