# Supplementary material for: Impact of Drying Processes on Phenolics and In Vitro Health-Related Activities of Indigenous Plants in Thailand
Source: Plants (Basel). 2022 Jan 22;11(3):294. doi: 10.3390/plants11030294 (PMC8838347; doi:10.3390/plants11030294)
Supplement: Supplementary file 1 [file plants-11-00294-s001.zip › plants-1532802-supplementary.pdf]

plants

Supplementary materials

Impact of Drying Processes on Phenolics and In Vitro Health-Related Activities of Indigenous Plants in Thailand

Pandaree Sirichai [1], Suwapat Kittibunchakul [1], Sirinapa Thangsiri [1], Nattira On-Nom [1], Chaowanee Chupeerach [1], Piya Temviriyanukul [1], Woorawee Inthachat [1], Onanong Nuchuchua [2], Amornrat Aursalung [1], Yuraporn Sahasakul [1], Somsri Charoenkiatkul [1] and Uthaiwan Suttisansanee [1,*]

[1] Institute of Nutrition, Mahidol University, Salaya, Phuttamonthon, Nakhon Pathom 73170, Thailand; riceberrii3@gmail.com (P.S.); suwapat.kit@mahidol.ac.th (S.K.); sirinapa.thang@outlook.com (S.T.); nattira.onn@mahidol.ac.th (N.O.-N.); chaowanee.chu@mahidol.ac.th (C.C.); piya.tem@mahidol.ac.th (P.T.); woorawee.int@mahidol.ac.th (W.I.); amornrat.aur@mahidol.ac.th (A.A.); yuraporn.sah@mahidol.ac.th (Y.S.); somsri.chr@mahidol.ac.th (S.C.)

[2] National Nanotechnology Center (NANOTEC), National Science and Technology Development Agency (NSTDA), Klong Luang, Pathum Thani 12120, Thailand; onanong@nanotec.or.th

* Correspondence: uthaiwan.sut@mahidol.ac.th; Tel.: +66-(0)-2800-2380 (ext. 422)

Supplementary Table S1:

Fragment ions of twenty-four authentic standards of phenolics using liquid chromatography–electrospray ionization–tandem mass spectrometry (LC-ESI-MS/MS) in selective reaction monitoring (SRM) mode.

Compounds	Standards	Ion mass	Parent ions (*m/z*)	SRM transitions (*m/z*) and collision energy (*V*)	RF lens (*V*)
1	Epigallocatechin gallate	[M–H]	457.175	305.155 (16.84 V), 168.97 (17.59 V), 125.042 (40.30 V)	204
2	Gallic acid	[M–H]	169.05	124.988 (14.56 V), 96.917 (18.77 V), 79.185 (22.94 V)	147
3	3,4-Dihydroxybenzoic acid	[M–H]	152.95	109.113 (14.35 V), 81.042 (20.50 V), 91.042 (24.59 V)	128
4	Chlorogenic acid	[M–H]	353.075	179.042 (14.06 V), 191.000 (16.54 V), 85.095 (39.96 V)	148
5	4-Hydroxybenic acid	[M–H]	137.05	92.970 (14.86 V), 65.000 (29.39 V), 75.000 (31.96 V)	110
6	Caffeic acid	[M–H]	179.038	135.054 (15.07 V), 107.071 (22.57 V), 85.042 (31.96 V)	151
7	Syringic acid	[M–H]	197.138	182.185 (13.72 V), 167.113 (19.24 V), 123.095 (22.31 V)	130
8	Vanillic acid	[M–H]	167.000	123.042 (11.66 V), 151.97 (14.59 V), 108.042 (18.65 V)	114
9	*p*-Coumaric acid	[M+H]	165.05	147.054 (11.70 V), 119.113 (19.36 V), 91.125 (25.89 V)	90
10	Rutin	[M+H]	611.20	303.13 (20.80), 465.20 (12.71V)	198
11	Sinapic acid	[M–H]	223.25	208.125 (13.51 V), 164.024 (15.78 V), 192.970 (22.65 V)	141
12	Ferulic acid	[M–H]	192.95	149.125 (11.28 V), 177.970 (13.05 V), 134.042 (16.50 V)	124
13	Hesperidin	[M–H]	609.30	301.179 (24.50 V), 325.179 (27.83 V), 286.125 (41.60 V)	299
14	Myricetin	[M–H]	317.088	178.970 (19.53 V), 150.988 (24.50 V), 137.113 (26.86 V)	245
15	Rosmarinic acid	[M–H]	359.20	197.000 (15.70 V), 161.113 (17.38 V), 133.054 (37.81 V)	175
16	Luteolin	[M–H]	285.138	197.000 (15.70 V), 161.113 (17.38 V), 133.054 (37.81 V)	241
17	Quercetin	[M–H]	301.200	178.976 (18.18 V), 273.125 (19.45 V), 151.042 (21.39 V)	237
18	Cinnamic acid	[M–H]	147.00	103.00 (11.23V), 77.083 (23.07)	107
19	Apigenin	[M–H]	269.075	116.863 (34.28 V), 149.071 (25.13 V), 151.131 (25.05 V)	244
20	Genistein	[M–H]	269.138	224.054 (25.60 V), 159.054 (29.26 V), 132.929 (30.95 V)	239
21	Naringenin	[M+H]	272.938	146.97 (21.01 V), 153.054 (24.42 V), 119.000 (31.28 V)	160

Supplementary Table S1 (Cont.):

Fragment ions of twenty-four authentic standards of phenolics using liquid chromatography–electrospray ionization–tandem mass spectrometry (LC-ESI-MS/MS) in selective reaction monitoring (SRM) mode.

Compounds	Standards	Ion mass	Parent ions (*m*/*z*)	SRM transitions (*m*/*z*) and collision energy (*V*)	RF lens (*V*)
22	Kaempferol	[M–H]	285.150	184.911 (25.85 V), 239.113 (27.03 V), 186.988 (28.17 V)	260
23	Isorhamnetin	[M–H]	315.088	300.000 (21.30 V), 150.970 (29.14 V), 271.054 (30.57 V)	233
24	Galangin	[M+H]	271.088	165.042 (28.80 V), 197.125 (31.75 V), 153.113 (32.42 V)	248

Supplementary Table S2:

The validation parameters of twenty-four authentic standards of phenolics using liquid chromatography–electrospray ionization–tandem mass spectrometry (LC-ESI-MS/MS) in selective reaction monitoring (SRM) mode.

Compounds	Retention time (min)	Standards	Linear range (µg/mL)	Linear regression equation	Correlation coefficient (R^2)	LOD (µg/mL)	LOQ (µg/mL)	%RSD (Inter-day)	%Recovery		
									Low level (µg/mL)	Medium level (µg/mL)	High level (µg/mL)
1	0.44	Epigallocatechin gallate	0.125–40	y =8533x + 1053.4	0.9985	0.067	0.230	0.023	91.84	85.36	91.37
2	0.564	Gallic acid	0.195–25	y =3323.1x – 2100.4	0.9984	0.04	0.14	0.01	113.05	118.57	109.12
3	0.803	3,4-Dihydroxybenzoic acid	0.195–25	y =11490x – 10877	0.9935	0.010	0.034	0.003	90.59	85.75	89.75
4	0.922	Chlorogenic acid	0.3125–40	y =8377.5x – 3623.5	0.9934	0.017	0.055	0.006	91.94	87.50	95.02
5	1.16	4-Hydroxybenic acid	0.3125–40	y =2482.6x– 3998.4	0.9917	0.027	0.090	0.009	109.67	103.60	101.28
6	1.40	Caffeic acid	0.3125–40	y =12328x–19725	0.9918	0.010	0.035	0.003	105.36	93.98	87.41
7	1.539	Syringic acid	3.125–100	y =68.091x + 230.43	0.9955	0.582	1.939	0.194	116.35	97.42	94.91
8	1.63	Vanillic acid	2.5–100	y =213.67x – 975.72	0.9900	0.15	0.48	0.05	99.86	101.76	100.12
9	2.452	*p*-Coumaric acid	0.3125–40	y =8532.4x–13559	0.9910	0.013	0.042	0.004	88.22	81.36	98.05
10	2.737	Rutin	0.009–1.25	y =49729x – 33.064	0.9999	0.001	0.005	0.0005	94.63	114.00	108.73
11	2.772	Sinapic acid	0.39–25	y =1592.6x – 832.22	0.9977	0.026	0.086	0.009	81.34	92.16	84.22
12	2.851	Ferulic acid	1.56–100	y =559.03x – 1819.2	0.9947	0.155	0.518	0.052	91.51	89.24	93.10
13	3.41	Hesperidin	0.25–40	y =838.63x – 242.2	0.9986	0.07	0.22	0.02	100.43	104.06	108.60
14	3.431	Myricetin	1.25–40	y =303.47x – 601.81	0.9976	0.261	0.871	0.087	113.07	81.77	91.12
15	3.528	Rosmarinic acid	0.3125–40	y =4322.4x-3744.1	0.9956	0.07	0.25	0.02	92.45	106.35	99.62
16	4.158	Luteolin	0.195–12.5	y =8381.9x – 5000.7	0.9945	0.015	0.050	0.0005	84.21	96.21	107.09
17	4.185	Quercetin	0.05–12.5	y =2934x + 917.17	0.9937	0.05	0.18	0.02	83.36	115.06	95.74

Supplementary Table S2 (Cont.):

The validation parameters of twenty-four authentic standards of phenolics using liquid chromatography–electrospray ionization–tandem mass spectrometry (LC-ESI-MS/MS) in selective reaction monitoring (SRM) mode.

Compounds	Retention time (min)	Standards	Linear range (µg/mL)	Linear regression equation	Correlation coefficient (R^2)	LOD (µg/mL)	LOQ (µg/mL)	%RSD (Inter-day)	%Recovery		
									Low level (µg/mL)	Medium level (µg/mL)	High level (µg/mL)
18	4.522	Cinnamic acid	0.039–10	y =6631.9x – 866.59	0.9964	0.049	0.163	0.016	101.94	98.84	95.85
19	4.689	Apigenin	0.34–11	y =1790.7x – 287.7	0.9997	0.127	0.424	0.042	88.84	106.89	114.79
20	4.693	Genistein	0.625–40	y =1247.2x – 1747.1	0.9977	0.049	0.163	0.016	95.33	101.49	11633
21	4.705	Naringenin	0.0008–5	y =16755x + 443.03	0.9932	0.003	0.011	0.001	117.92	96.26	111.08
22	4.79	Kaempferol	0.25–10	y =1006.8x – 346.28	0.9905	0.122	0.406	0.041	92.35	107.69	102.17
23	4.878	Isorhamnetin	0.0098–2.5	y =12698x + 586.16	0.9945	0.016	0.052	0.005	113.57	105.88	111.14
24	6.146	Galangin	0.3125–40	y =5012.1x – 9354.7	0.9879	0.010	0.035	0.003	84.01	112.92	115.80

Supplementary Table S3:

The scientific name, voucher specimen, folk medicine, plant biology and characterization, and physical appearance of the edible part of the plant samples.

Scientific name/voucher specimen/edible parts/ folk medicine	Plant biology/characterization	Physical appearance of the edible part
***Albizia lebbeck* (L.) Benth**<br><br>**Voucher specimens:** PBM-005664<br>**Edible part:** Shoot and young leaves<br>**Folk medicine [1]:** Leaves are used to cool the body and extinguish heated poisons.	A medium-sized deciduous perennial tree, which grows to a height of 3-15 meters in plantations and up to 30 meters in the open. Bipinnate leaves have 3-11 pairs of brilliant green, oblong leaflets that are 1.5-6.5 cm long and 0.5-3.5 cm wide. Inflorescences are globular clusters of 15-40 fragrant white flowers in a globular shape. The fruits are reddish-brown pods with 5-15 flat rounded, free moving seeds that are 10-30 cm long x 3-6 cm wide [12].	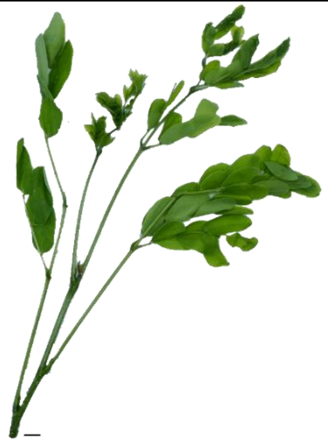<br>Young leaves and tender tips, light green color, and soft texture
***Alpinia malaccensis* (Burm.) Roscoe**<br><br>**Voucher specimens:** PBM-005663<br>**Edible part:** Rhizome<br>**Folk medicine [2]:** Underground rhizomes or stems have a spicy and bitter flavor and are used to treat gas, indigestion, colic, and as a carminative in women after childbirth. It is applied topically to relieve urticaria itching, joint discomfort and swelling, bruising, eczema, and chloasma.	This perennial-herbaceous plant has white, pungent, hot cinnamon-like scents rhizomes. Single leaves are arranged in a zigzag pattern, with edges that are parallel. White sub-flowers form a bouquet at the apex of 15-30 cm long blossoms, and the fruit is spherical once dried and can be broken out [13].	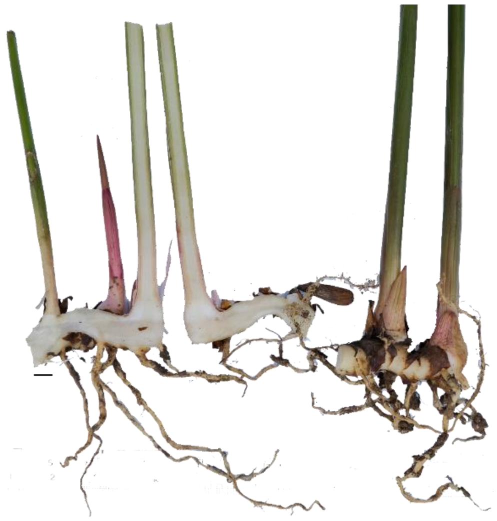<br>Underground rhizomes, white color
***Careya arborea* Roxb.**<br><br>**Voucher specimens:** PBM-005656<br>**Edible part:** Shoot and young leaves<br>**Folk medicine [3, 4]:** Young leaves and tender tips are used to treat flatulence.	Medium-sized deciduous perennial tree with many branches and small stems. The leaves are placed in a cluster at the terminal of the branches in an upside-down oval form. The flower is a bouquet with four white petals, bell-shaped base sepals, and a circular green fruit with many seeds [14].	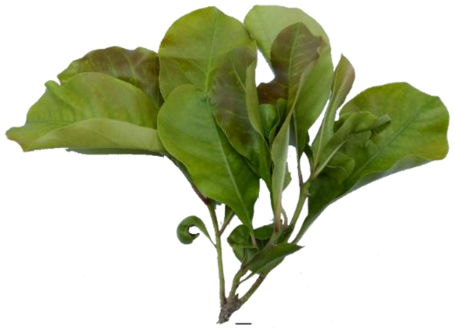<br>Young leaves and tender tips, light green and purple color, soft texture of the leaves

Note: The (—) indicates the scale of 1 cm.

Supplementary Table S3 (Cont.):

The scientific name, voucher specimen, and physical appearance of the edible part of the samples.

Scientific name/voucher specimen/edible parts/ folk medicine	Plant biology/characterization	Physical appearance of the edible part
***Diplazium esculentum* (Retz.)**<br><br>**Voucher specimens:** PBM-005654<br>**Edible part:** Shoot and young leaves<br>**Folk medicine [5]:** A decoction of *D.esculentum* is used by women as a tonic after childbirth and can be used to cure expectoration of blood and ordinary coughs.	This perennial vegetable fern has a creeping rhizome and thick black roots growing beneath the surface. Spirally borne leaves reach a height of 1 m or more and are compactly placed. Rhizome scales are around 1 cm long and have a narrow, tapering toothed shape. The pinnules are lanceolate, 5 cm long, and coarsely serrated. The leaves are 2- or 3-pinnate and 50 to 80 cm long [15].	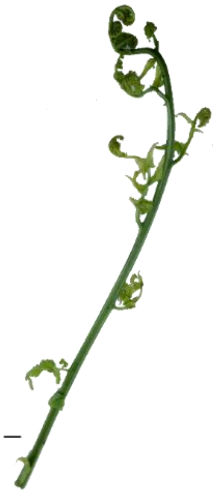<br>Tender curling leaves, fresh green color
***Kaempferia roscoeana* Wall.**<br><br>**Voucher specimens:** PBM-005658<br>**Edible part:** Shoot and young leaves<br>**Folk medicine [6]:** No information on shoots and young leaves; however, rhizomes are used to pound and mask on the wound to neutralize insect bites.	The perennial herbaceous plant with the small stem has only two leaves that are spread flat on the ground. The leaf is fairly spherical, green in hue, and bears dark green stripes. The blooms are white, and the petals of the white petals are pale yellow at the base of the petals, bunched between the leaf sheath [16].	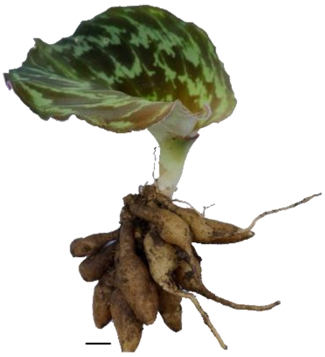<br>Fairly spherical leaf, green in hue, and bears dark green/purple stripes
***Millettia brandisiana* Kurz.**<br><br>**Voucher specimens:** PBM-005652<br>**Edible part:** Shoot and young leaves<br>**Folk medicine [7]:** No information on young leaves and tender tips; however, trunk is boiled with water and drink to nourish the blood, while bark is boiled with water used to cleanse chronic wounds. Heartwood is used as a tonic for blood, body nourishment, and improving appetite.	A deciduous tree that grows to be 10-20 meters tall, minimally branching, and smooth. Feather-like leaves. Blooms with a lot of purple-pink petals, similar to pea flowers. Oval flat pods are 2-2.5 cm wide and 7-8 cm long with pointy or rounded tip, thin hairs, and dark red-brown seeds [7].	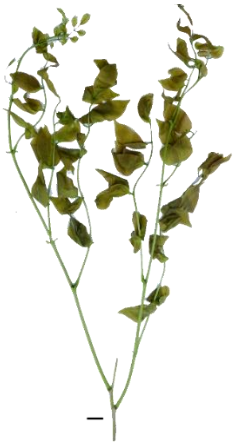<br>Young leaves and tender tips, light green and purple color

Note: The (—) indicates the scale of 1 cm.

Supplementary Table S3 (Cont.):

The scientific name, voucher specimen, and physical appearance of the edible part of the samples.

Scientific name/voucher specimen/edible parts/ folk medicine	Plant biology/characterization	Physical appearance of the edible part
Momordica charantia<br><br>**Voucher specimens:** PBM-005659<br>**Edible part:** Whole fruit<br>**Folk medicine [8]:** Unripe fruits have a bitter flavor and can be used to treat fever (boil with water and drink). They are also useful for diabetes, nourishing elements, blemish mouth, nourishing bile, good appetite, nourishing menstrual blood and used as a moderate laxative.	This plant is a creeper with a single leaf, arranged in the alternate. The leaf shape is like a palm, wide-long 4-7 cm, with the edge of the concave around 5-7 deep edge. Flowers have yellow bell-shaped petals, while the fruit is fresh, bobbin-shaped, bumpy, and bitter [17].	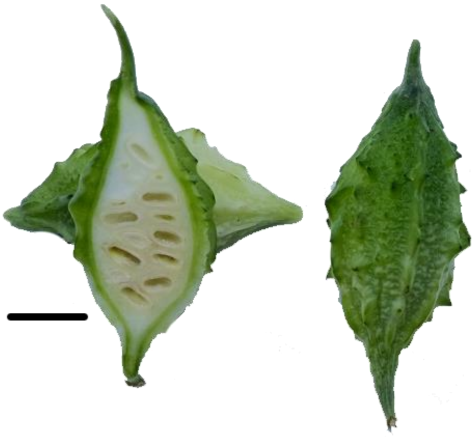<br>Young, green, unripe fruits
Phyllanthus emblica **L.**<br><br>**Voucher specimens:** PBM-005661<br>**Edible part:** Flesh of the fruit (no seed)<br>**Folk medicine [9]:** Ripe fruits have sour-astringent taste, which can reduce fever, diuretic, cough, cure phlegm, and moisten the throat. They can also nourish the heart, purify the blood, cure wind, and cure scurvy.	This deciduous tree has small to medium size. The leaves are simple, subsessile, and closely set along branchlets with light green color. The flowers are greenish-yellow. The fruit is nearly spherical, light greenish-yellow, with six vertical stripes or furrows. The taste of the fruit is sour, bitter, and astringent [18].	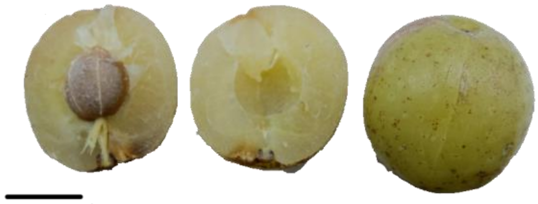<br>Ripe fruits, nearly spherical, light greenish-yellow, with six vertical stripes or furrows
Zingiber cassumunar **Roxb.**<br><br>**Voucher specimens:** PBM-005665<br>**Edible part:** Rhizome<br>**Folk medicine [10]:** Rhizomes or underground stems have astringent taste. It is a remedy for flatulence, indigestion, has a mild laxative effect, heals the intestines, cures toxins in the stomach, cures asthma, expels gas in the intestines, cures diarrhea, is used as a drug to help menstruate women after childbirth, reduces inflammation and swelling, cures diarrhea, relieves toothache, and cures vomiting blood. Fresh rhizomes are used as external medications, mashed to make oil, and used to treat beriberi, aches and pains, sprains, and swelling.	This perennial-herbaceous plant has tuberous furnished with long, white fleshy fibers and jointed like ginger but much larger. When fresh, it has a deep yellow color with a strong camphorated odor and a warm spicy, bitterest taste [19].	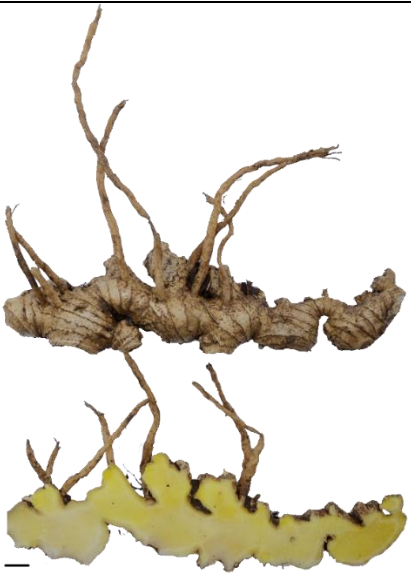<br>Fresh rhizomes, yellow color inside, possessing a strong camphorated odor

Note: The (—) indicates the scale of 1 cm.

Supplementary Table S3 (Cont.):

The scientific name, voucher specimen, and physical appearance of the edible part of the samples.

Scientific name/voucher specimen/edible parts/ folk medicine	Plant biology/characterization	Physical appearance of the edible part
Zingiber citriodorum **J.Mood & T. Theleide Zingiber**<br><br>**Voucher specimens:** PBM-005674<br>**Edible part:** Rhizome<br>**Folk medicine [11]:** Tubers can use as an ingredient for making chili paste. They can carminative and relieve flatulence.	This perennial-herbaceous plant has the underground rhizomes, which are yellow. Stems above the soil are about 50 cm high. The whole tree smells like lemon. The leaves are oblong, green, while the dorsal side is dark gray. The inflorescence is caused by rhizomes. An upright bouquet and the leaves are light green, but will turn red when aged [20].	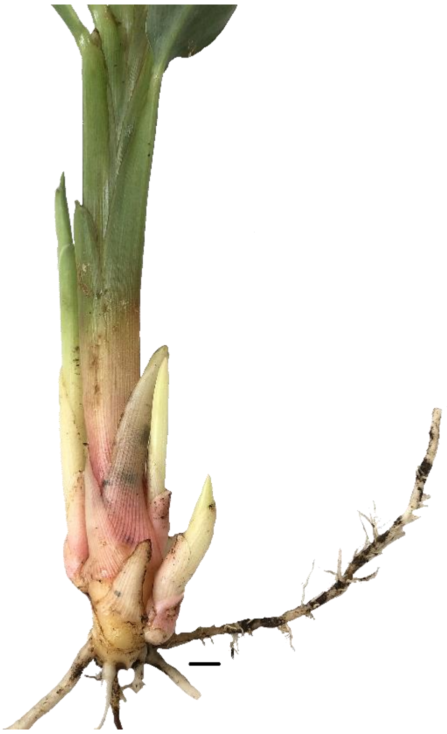<br>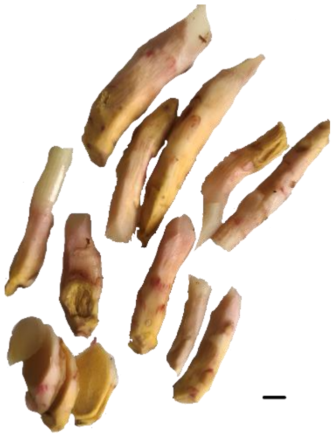<br>Underground tubers, yellow color inside, covering with pink peel

Note: The (—) indicates the scale of 1 cm.

Supplementary Table S4:

The percentage of moisture content in edible part of fresh, freeze-dried and oven-dried plant samples.

Samples	Moisture content (%)		
	Fresh	Freeze-dry	Oven-dry
Albizia lebbeck (L.) Benth	72.3 ± 0.9	4.5 ± 0.3	5.5 ± 0.4
Alpinia malaccensis (Burm.) Roscoe	36.5 ± 1.6	5.6 ± 0.1	5.7 ± 0.1
Careya arborea Roxb.	35.2 ± 0.5	6.5 ± 0.1	4.4 ± 0.1
Diplazium esculentum (Retz.) Swartz	74.9 ± 0.4	4.7 ± 0.2	6.7 ± 0.2
Kaempferia roscoeana Wall.	83.1 ± 1.2	6.7 ± 0.1	4.1 ± 0.1
Millettia brandisiana Kurz.	70.9 ± 0.5	4.3 ± 0.3	5.8 ± 0.3
Momordica charantia	37.6 ± 1.5	6.9 ± 0.1	NA
Phyllanthus emblica L.	82.0 ± 0.4	3.5 ± 0.4	3.2 ± 0.3
Zingiber cassumunar Roxb.	69.6 ± 2.3	6.8 ± 0.1	6.9 ± 0.1
Zingiber citriodorum J.Mood & T. Theleide	73.2 ± 1.4	7.0 ± 0.2	7.9 ± 0.1

N/A : Not available due to limited quantity of the sample.

Supplementary Figure S1:

The liquid chromatography–electrospray ionization–tandem mass spectrometry (LC-ESI-MS/MS) chromatograms of twenty-four authentic standards of phenolics.

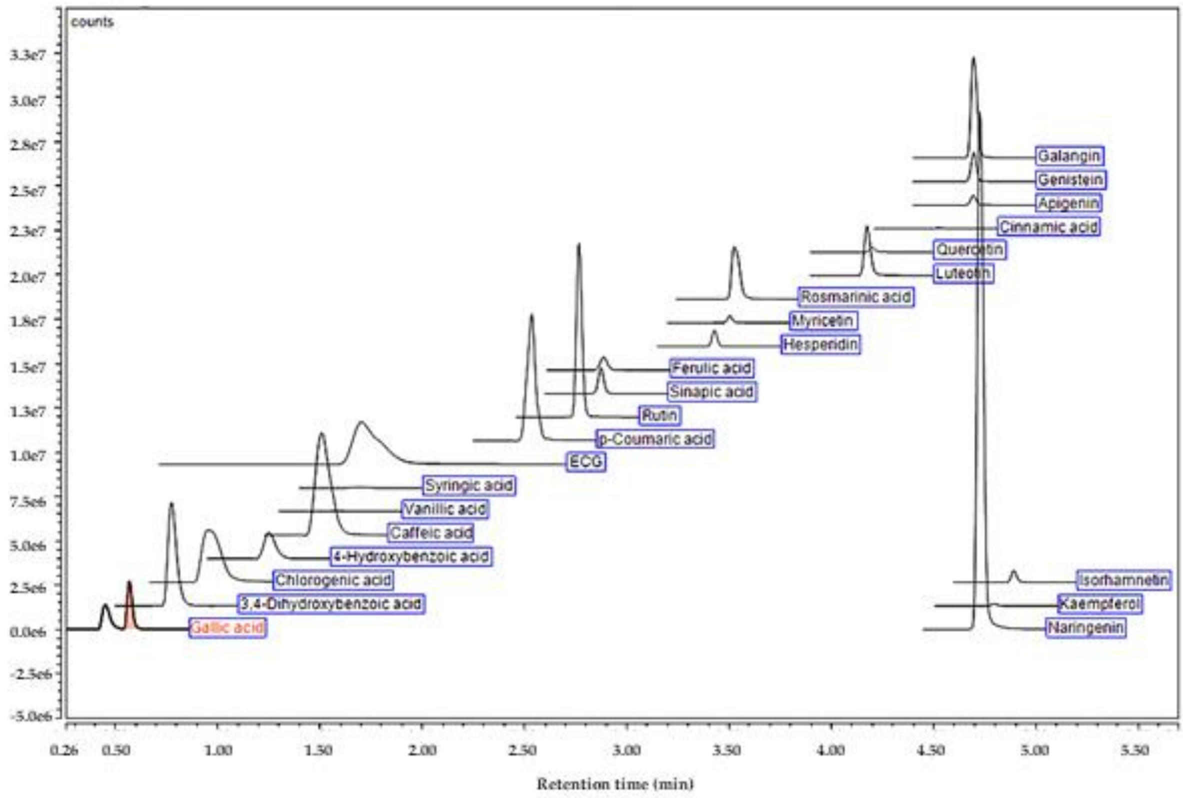

Supplementary Figure S2:

The liquid chromatography–electrospray ionization–tandem mass spectrometry (LC-ESI-MS/MS) chromatograms of ten plant samples including (**a**) *Albizia lebbeck* (L.) Benth, (**b**) *Alpinia malaccensis* (Burm.) Roscoe, (**c**) *Careya arborea* Roxb., (**d**) *Diplazium esculentum* (Retz.) Swartz, (**e**) *Kaempferia roscoeana* Wall., (**f**) *Millettia brandisiana* Kurz., (**g**) *Momordica charantia*, (**h**) *Phyllanthus emblica* L., (**i**) *Zingiber cassumunar* Roxb., and (**j**) *Zingiber citriodorum* J.Mood & T. Theleide.

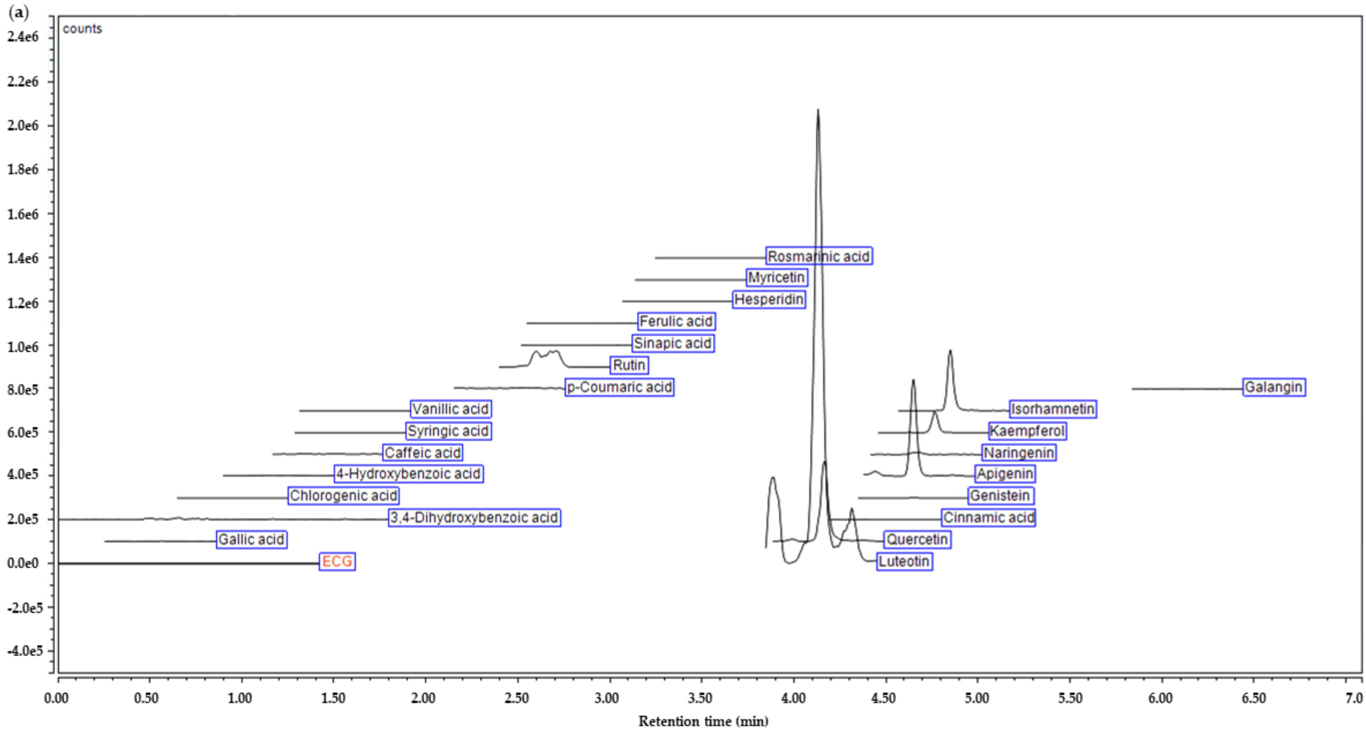

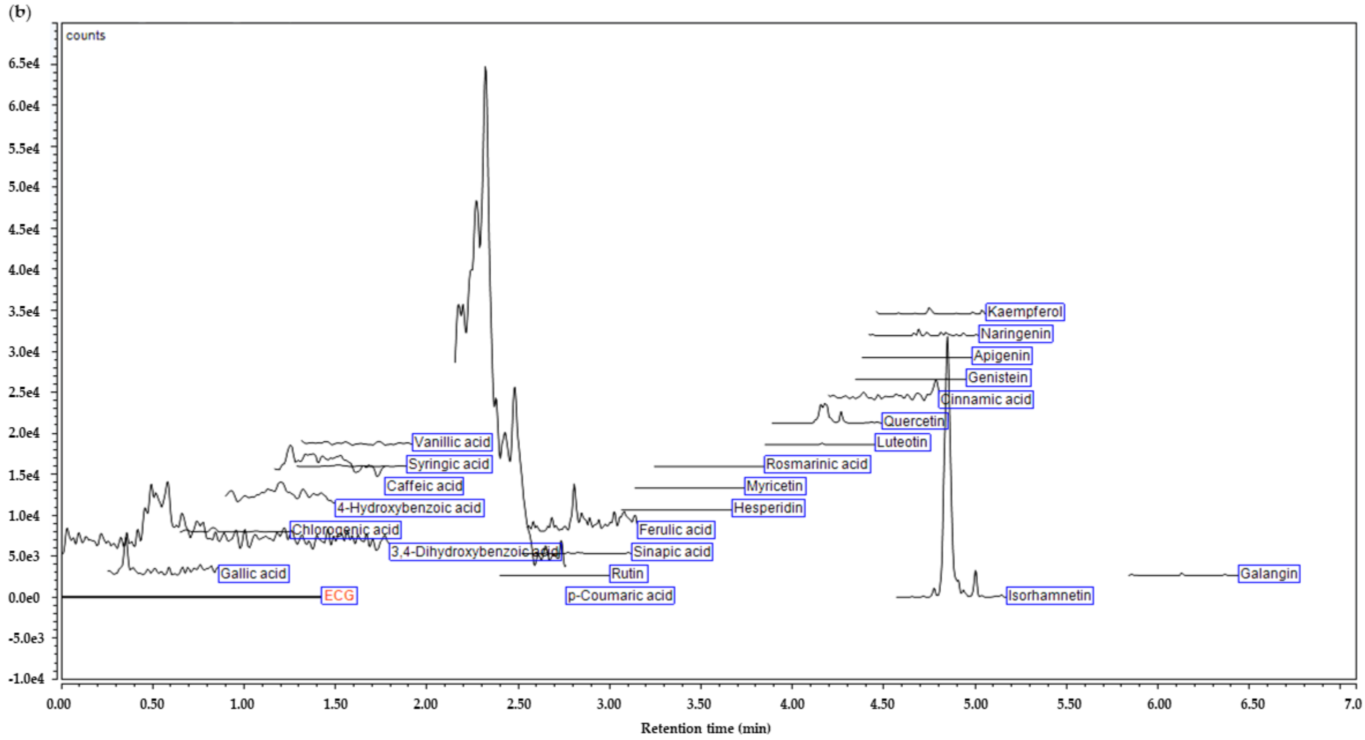

Supplementary Figure S2 (Cont.):

The liquid chromatography–electrospray ionization–tandem mass spectrometry (LC-MS/MS) chromatograms of ten plant samples including (**a**) *Albizia lebbeck* (L.) Benth, (**b**) *Alpinia malaccensis* (Burm.) Roscoe, (**c**) *Careya arborea* Roxb., (**d**) *Diplazium esculentum* (Retz.) Swartz, (**e**) *Kaempferia roscoeana* Wall., (**f**) *Millettia brandisiana* Kurz., (**g**) *Momordica charantia*, (**h**) *Phyllanthus emblica* L., (**i**) *Zingiber cassumunar* Roxb., and (**j**) *Zingiber citriodorum* J.Mood & T. Theleide.

(c)

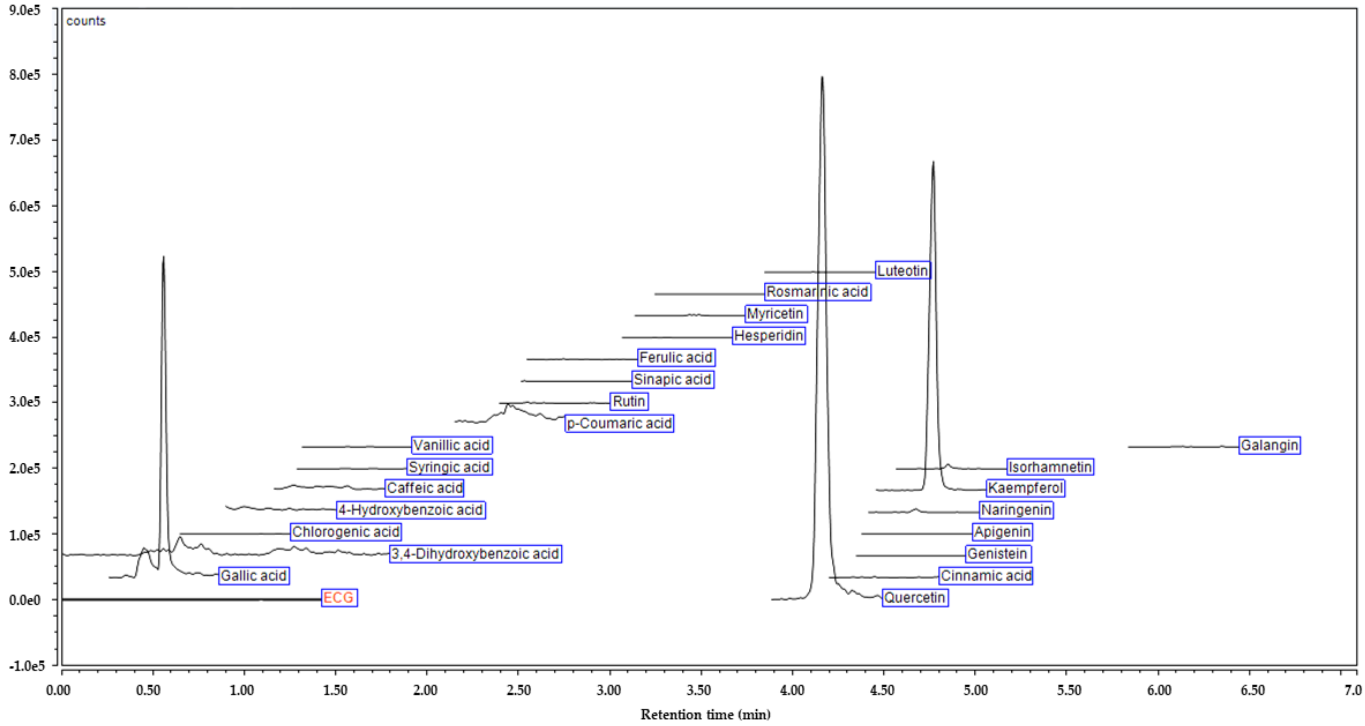

(d)

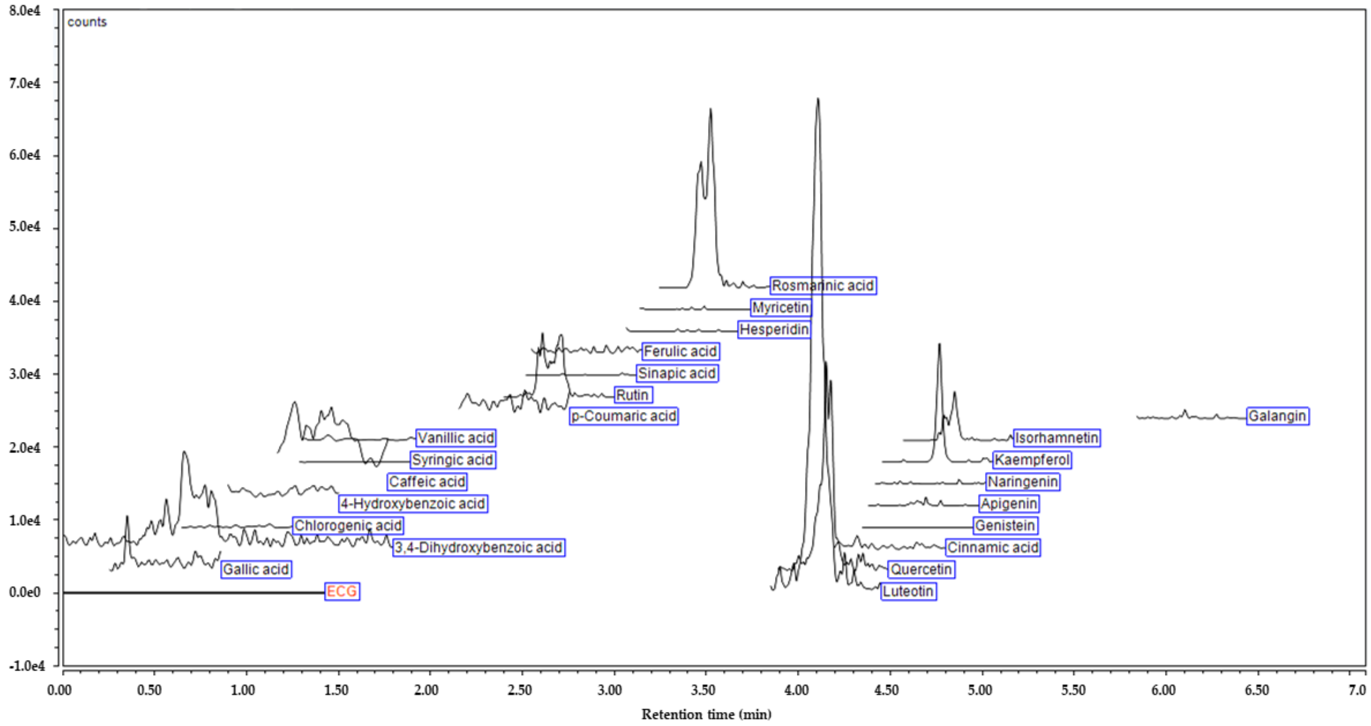

Supplementary Figure S2 (Cont.):

The liquid chromatography–electrospray ionization–tandem mass spectrometry (LC-MS/MS) chromatograms of ten plant samples including (**a**) *Albizia lebbeck* (L.) Benth, (**b**) *Alpinia malaccensis* (Burm.) Roscoe, (**c**) *Careya arborea* Roxb., (**d**) *Diplazium esculentum* (Retz.) Swartz, (**e**) *Kaempferia roscoeana* Wall., (**f**) *Millettia brandisiana* Kurz., (**g**) *Momordica charantia*, (**h**) *Phyllanthus emblica* L., (**i**) *Zingiber cassumunar* Roxb., and (**j**) *Zingiber citriodorum* J.Mood & T. Theleide.

(e)

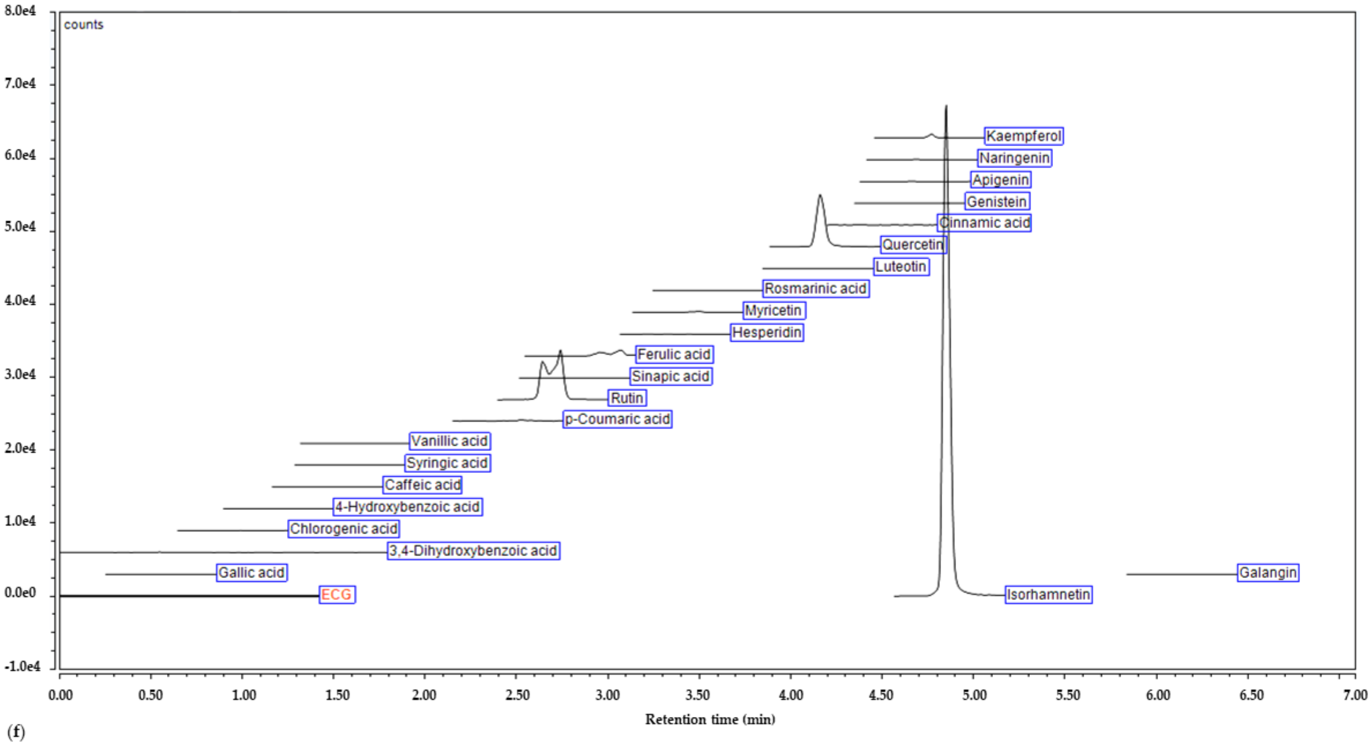

(f)

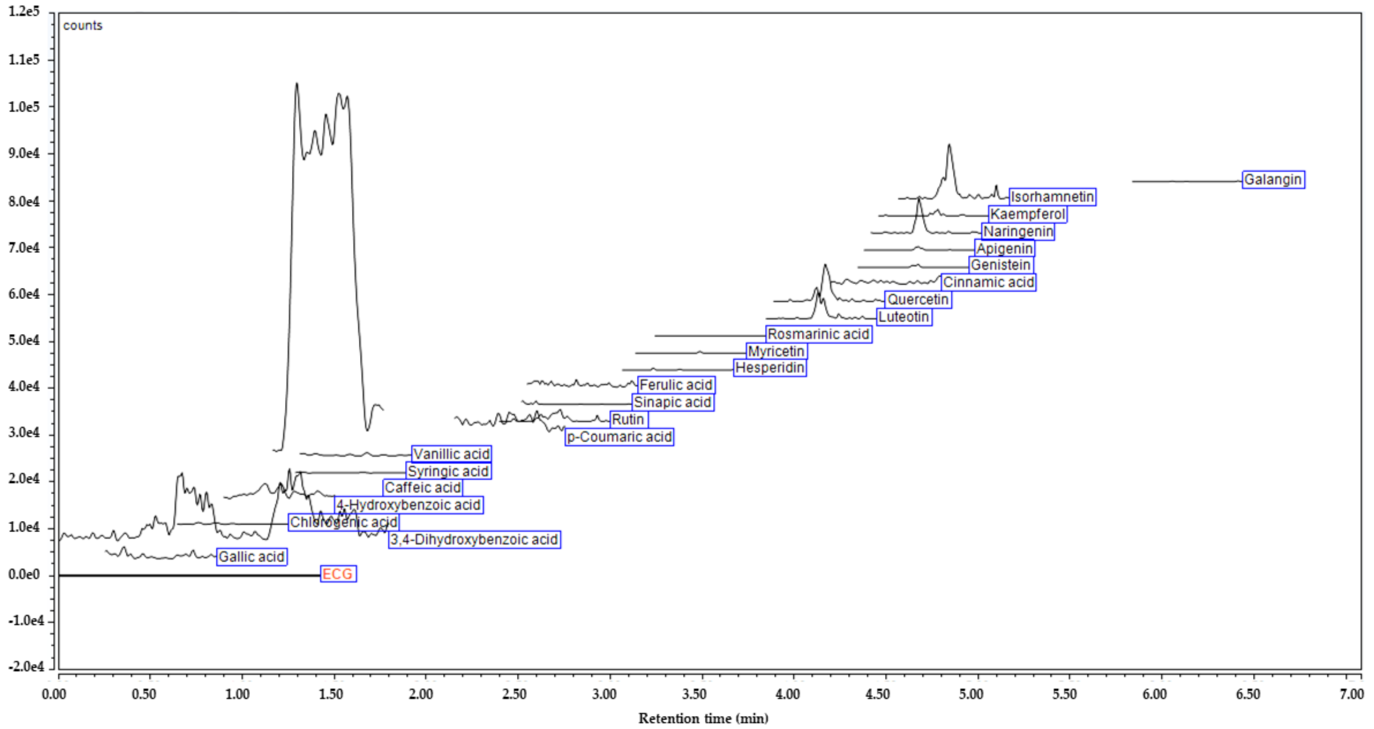

Supplementary Figure S2 (Cont.):

The liquid chromatography–electrospray ionization–tandem mass spectrometry (LC-MS/MS) chromatograms of ten plant samples including (**a**) *Albizia lebbeck* (L.) Benth, (**b**) *Alpinia malaccensis* (Burm.) Roscoe, (**c**) *Careya arborea* Roxb., (**d**) *Diplazium esculentum* (Retz.) Swartz, (**e**) *Kaempferia roscoeana* Wall., (**f**) *Millettia brandisiana* Kurz., (**g**) *Momordica charantia*, (**h**) *Phyllanthus emblica* L., (**i**) *Zingiber cassumunar* Roxb., and (**j**) *Zingiber citriodorum* J.Mood & T. Theleide.

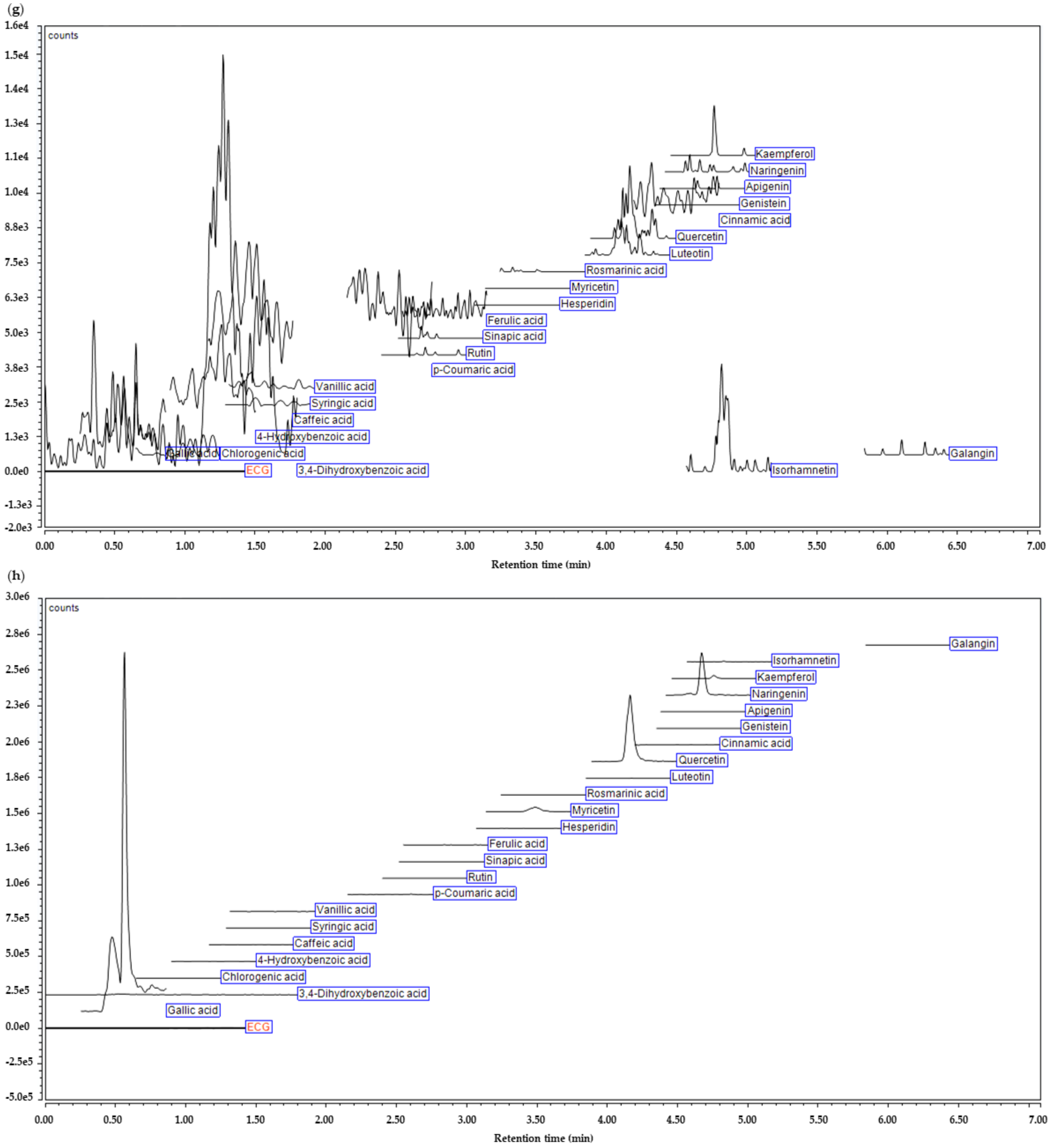

Supplementary Figure S2 (Cont.):

The liquid chromatography–electrospray ionization–tandem mass spectrometry (LC-MS/MS) chromatograms of ten plant samples including (**a**) *Albizia lebbeck* (L.) Benth, (**b**) *Alpinia malaccensis* (Burm.) Roscoe, (**c**) *Careya arborea* Roxb., (**d**) *Diplazium esculentum* (Retz.) Swartz, (**e**) *Kaempferia roscoeana* Wall., (**f**) *Millettia brandisiana* Kurz., (**g**) *Momordica charantia*, (**h**) *Phyllanthus emblica* L., (**i**) *Zingiber cassumunar* Roxb., and (**j**) *Zingiber citriodorum* J.Mood & T. Theleide.

(i)

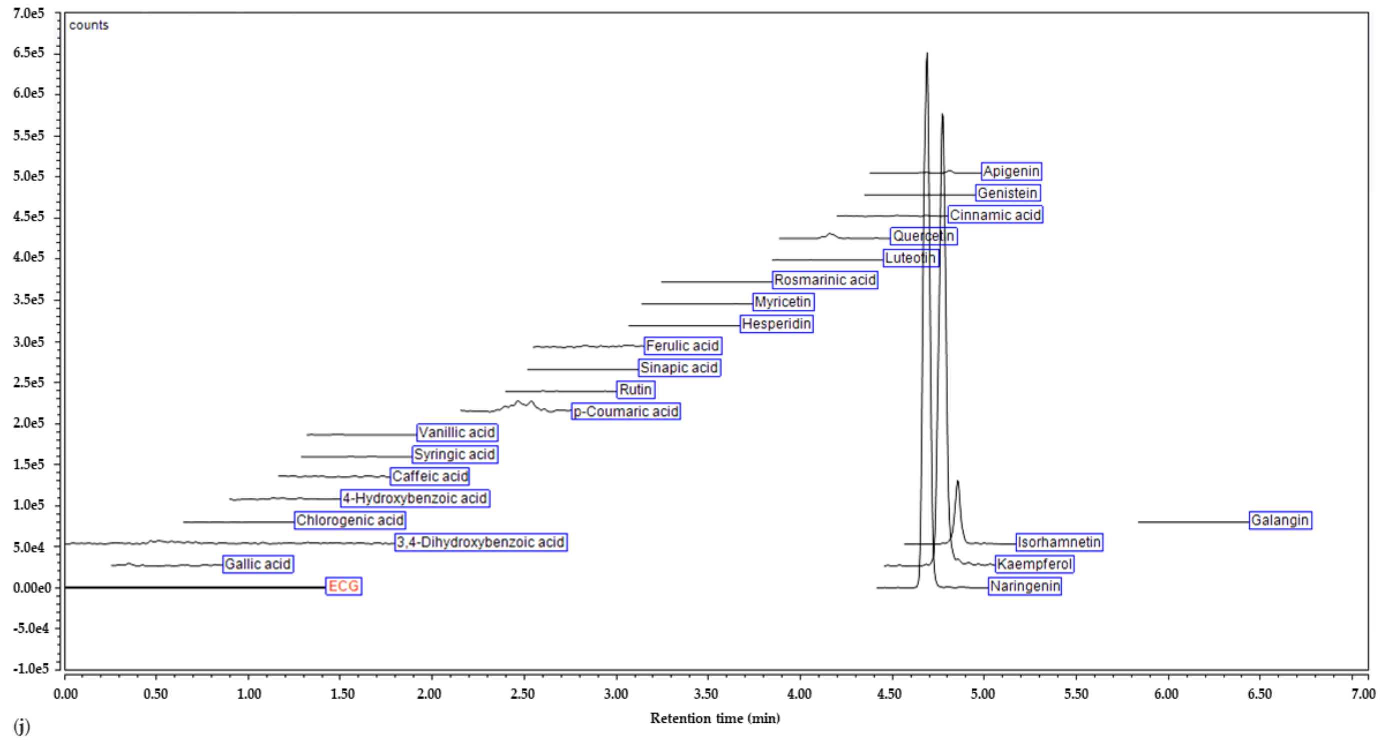

(j)

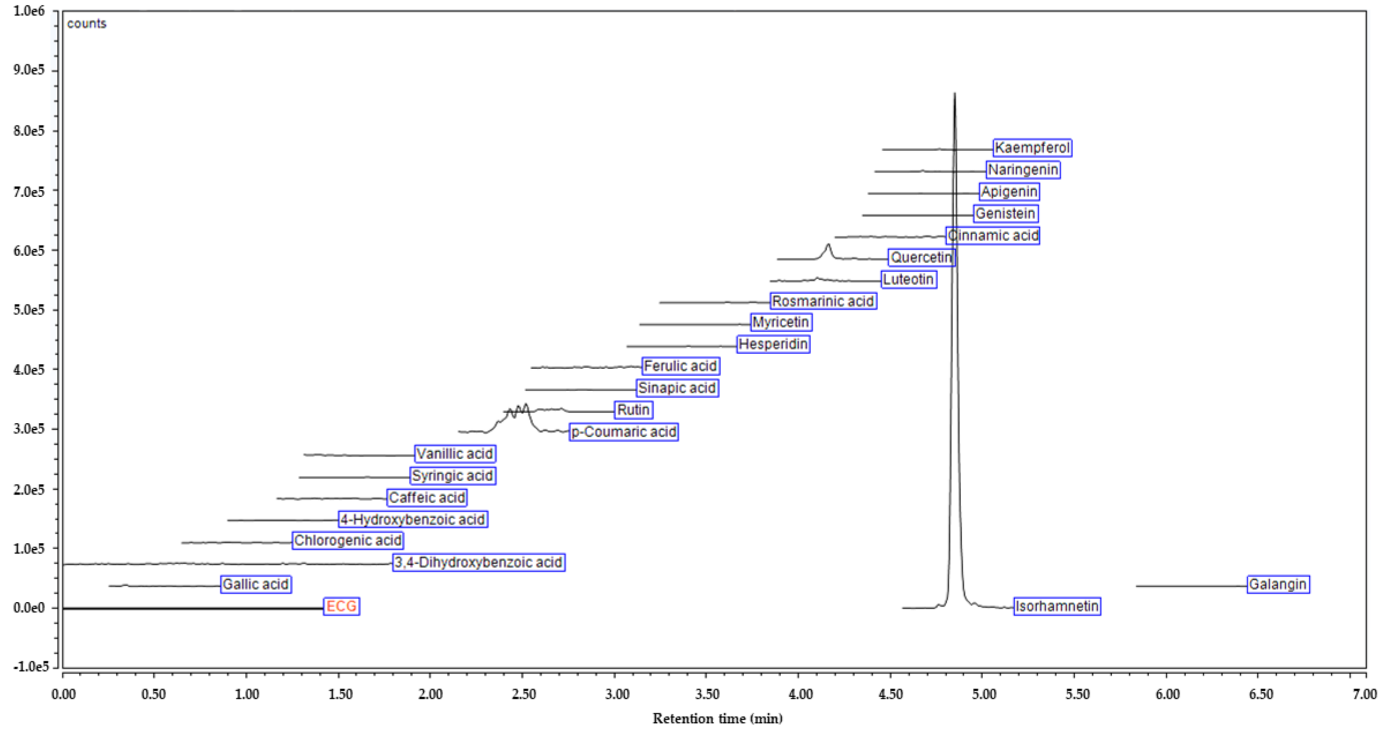

References

1. Samunprai.com. Herb database "Albizia lebbeck (Linn.) Benth". 2021. Available from: https://www.samunpri.com/พฤกษ์/.
2. Medicinal plant information, F.o.P., Silpakorn University. Alpinia galanga (L.) Willd. 2021; Available from: http://pharmacy.su.ac.th/herbmed/herb/text/herb_detail.php?herbID=56.
3. The botanical garden organization, M.o.N.R.a.E., Thailand. BGO Plant databases"Careya arborea Roxb.". 2021 [cited 2021-30-12]; Available from: http://www.qsbg.org/database/Botanic_Book%20full%20option/search_detail.asp?botanic_id=2858.
4. Medicinal plant information, F.o.P., Silpakorn University. Careya sphaeice Roxb. 2021; Available from: http://pharmacy.su.ac.th/herbmed/herb/text/herb_detail.php?herbID=449.
5. De Winter, W.; Amoroso, V. Plant Resources of South-East Asia. Cryptogams: ferns and fern allies. 2003, 15(2).
6. Samunprai.com. Herb database "Milettia Brandisiana KURZ". 2021; Available from: https://www.samunpri.com/กระพี้จั่น/.
7. Medicinal plant information, F.o.P., Silpakorn University. Kaempferia roscoeana Wall. 2021; Available from: http://pharmacy.su.ac.th/herbmed/herb/text/herb_detail.php?herbID=375.
8. Medicinal plant information, F.o.P., Silpakorn University. Momordica charantia L. 2021; Available from: http://pharmacy.su.ac.th/herbmed/herb/text/herb_detail.php?herbID=182.
9. Medicinal plant information, F.o.P., Silpakorn University. Phyllanthus emblica L. 2021; Available from: http://pharmacy.su.ac.th/herbmed/herb/text/herb_detail.php?herbID=176.
10. Medicinal plant information, F.o.P., Silpakorn University. Zingiber cassumunar Roxb. 2021; Available from: http://pharmacy.su.ac.th/herbmed/herb/text/herb_detail.php?herbID=163.
11. Inthachoob, P. *Zingiber citriodorum* Theilade & Mood. Edible plants and Herb, Bangkok Herbarium (BK). 2018, 2.
12. Heuzé V.T.G.; Sauvant D. Lebbek (*Albizia lebbeck*). Feedipedia, a programme by INRA, CIRAD, AFZ and FAO. 2015, Available from: https://www.feedipedia.org/node/334.
13. The botanical garden organization, M.o.N.R.a.E., Thailand. BGO Plant databases"*Alpinia malaccensis*". 2017, Available from: http://www.qsbg.org/Database/Botanic_Book%20full%20option/Search_detail.asp?Botanic_ID=2845.
14. Sirindhorn, T.P.G.C.P.u.t.R.I.o.H.R.H.P.M.C. *Careya arborea* Roxb. Plant databases; Available from: http://www.rspg.or.th/plants_data/plantdat/lecythid/csphae_2.htm.
15. Sirindhorn, B.U.o.t.P.G.C.P.u.t.R.I.o.H.R.H.P.M.C. Plant databaese"*Diplazium esculentum* (Retz.) Sw". 2020, Available from: http://www.rspg.or.th/plants_data/plantdat/athyriac/descul_1.htm.
16. The botanical garden organization, M.o.N.R.a.E., Thailand. BGO Plant databases"*Kaempferia roscoeana* Wall.". 2017, Available from: http://www.qsbg.org/Database/Botanic_Book%20full%20option/search_detail.asp?botanic_id=2959.
17. Herbal Information Office, F.o.P., Mahidol University. "CUCURBITACEAE *Momordica charantia* L.". 2020, Available from: http://www.medplant.mahidol.ac.th/pharm/botanic.asp?bc=0527&kw=%C1%D0%C3%D0%A2%D5%E9%B9%A1*
18. Plants For A Future. Edible Shrubs: *Phyllanthus emblica* - L. 2020, Available from: https://pfaf.org/user/Plant.aspx?LatinName=Phyllanthus+emblica#:~:text=Emblic%20or%20Phyllanthus%20emblica%20is,about%2035%20cm%20in%20diameter.
19. Ethnobotany, Phytochemistry and Pharmacology of *Zingiber cassumunar* Roxb. (Zingiberaceae). 2020, Available from: https://www.researchgate.net/publication/287674414_Ethnobotany_Phytochemistry_and_Pharmacology_of_Zingiber_cassumunar_Roxb_Zingiberaceae.
20. The botanical garden organization, M.o.N.R.a.E., Thailand. BGO Plant databases"*Zingiber citriodorum* Theilade & Mood". 2017, Available from: http://www.qsbg.org/Database/Botanic_Book%20full%20option/search_detail.asp?Botanic_ID=2962.
